# Free Vibrations of Sandwich Plates with Damaged Soft-Core and Non-Uniform Mechanical Properties: Modeling and Finite Element Analysis

**DOI:** 10.3390/ma12152444

**Published:** 2019-07-31

**Authors:** Michele Bacciocchi, Raimondo Luciano, Carmelo Majorana, Angelo Marcello Tarantino

**Affiliations:** 1 Department of Civil, Chemical, Environmental, and Materials Engineering (DICAM), University of Bologna, Viale del Risorgimento, 40136 Bologna, Italy; 2 Dipartimento di Economia, Scienze e Diritto (DESD), University of San Marino, Via Consiglio dei Sessanta, 47891 Dogana, San Marino; 3 Engineering Department, University of Napoli Parthenope, Via Ammiraglio Ferdinando Acton, 80133 Napoli, Italy; 4 Department of Civil, Environmental and Architectural Engineering (DICEA), University of Padova, Via F. Marzolo, 35131 Padova, Italy; 5 Department of Engineering “Enzo Ferrari” (DIEF), University of Modena and Reggio Emilia, Via Vivarelli, 41125 Modena, Italy

**Keywords:** three-phase composite materials, Finite Element modeling, sandwich plates, zig-zag theory, carbon nanotubes, free vibrations

## Abstract

The paper aims to investigate the natural frequencies of sandwich plates by means of a Finite Element (FE) formulation based on the Reissner-Mindlin Zig-zag (RMZ) theory. The structures are made of a damaged isotropic soft-core and two external stiffer orthotropic face-sheets. These skins are strengthened at the nanoscale level by randomly oriented Carbon nanotubes (CNTs) and are reinforced at the microscale stage by oriented straight fibers. These reinforcing phases are included in a polymer matrix and a three-phase approach based on the Eshelby-Mori-Tanaka scheme and on the Halpin-Tsai approach, which is developed to compute the overall mechanical properties of the composite material. A non-uniform distribution of the reinforcing fibers is assumed along the thickness of the skin and is modeled analytically by means of peculiar expressions given as a function of the thickness coordinate. Several parametric analyses are carried out to investigate the mechanical behavior of these multi-layered structures depending on the damage features, through-the-thickness distribution of the straight fibers, stacking sequence, and mass fraction of the constituents. Some final remarks are presented to provide useful observations and design criteria.

## 1. Introduction

Since its early development and the publication of the first research papers [1,2,3,4,5,6], the Finite Element (FE) method has shown its potentiality in solving easily and accurately many structural problems which could not be solved analytically. Nowadays, this feature is even more emphasized by the great and continuous technological advancements reached in computer sciences in terms of available computational resources. As highlighted in the books which can be certainly considered as milestones in the development of the FE method [7,8,9,10,11,12,13], the easy implementation and the possibility to reduce complex continuous problems into simpler discrete ones has encouraged its rapid spread among many researchers and engineers [14,15,16,17,18,19,20].

In the current paper, the FE technique is implemented in a computational code to solve the free vibration analysis of three-layered sandwich plates with a damaged soft-core and non-uniform mechanical properties. In particular, the structures are made by an isotropic core that undergoes a progressive uniform damage, which is modeled as a decay of the mechanical properties expressed in terms of engineering constants. The damage model is the one illustrated in the book by Lemaitre and Chaboche [21], and it is representative of the formation of microcracks and discontinuities within the considered medium. Since these defects are uniformly distributed and affect the central layer of the plates independently from the direction, this phenomenon is known as “isotropic damage” and it is fully described by a scalar parameter. Further details concerning the phenomenological aspects related to the damage of materials can be found in the book by Reddy and Miravete [22], whereas some structural applications which investigate the effect of damage are illustrated in [23,24,25,26,27,28,29,30,31,32,33].

The soft-core of the structures is surrounded and strengthened by two external sinks (or face-sheets), which are stiffer and thinner than the central layer. These plies are made of a polymer matrix which contains randomly oriented Carbon nanotubes (CNTs) and keeps together straight Carbon fibers. The role of the matrix in composite materials is clearly illustrated in the books by Vinson [34], Jones [35], Reddy [36], and Barbero [37]. These works should be taken into account as complete references for the analysis and modelling of composite materials. As highlighted in the papers [38,39], the matrix is reinforced at different levels. At the nano-scale, the CNTs provide the matrix with additional stiffness [40,41,42,43,44,45,46,47,48,49]. The single fiber of Carbon nanotube (CNT) is modeled as a transversely isotropic cylinder as suggested by Odegard et al. [50] and it assumed to be randomly oriented in the matrix, following the approach developed in the paper by Shi et al. [51]. The Eshelby-Mori-Tanaka scheme is applied at this level to evaluate the mechanical properties of this enriched matrix [52,53], which has isotropic features due to the random orientation of CNTs [54]. On the other hand, the reinforcing phase at the micro-scale is given by oriented straight fibers. The semi-empirical Halpin-Tsai homogenization procedure [55,56,57], which is based on the use of the Hill’s elastic moduli [58,59], is employed at this level to obtain an accurate estimation of the overall mechanical features of the composite skins. Therefore, a multi-phase approach has been developed to this aim [60,61,62,63].

The development of micromechanics theories able to evaluate the mechanical features of fiber-reinforced composites has always fascinated many researchers, as illustrated in a complete manner by Chamis and Sendeckyj [64]. For completeness purposes, it should be recalled that different methods can be found in the literature as alternatives to the Halpin-Tsai semi-empirical homogenization procedure, such as self- consistent models [65,66], variational methodologies [67,68], and techniques based on the mechanics of materials [69,70,71]. Further details can be found also in the papers [72,73].

With respect to previous contributions, the straight fibers are graded in the thickness direction. In other words, the volume fraction distribution of the reinforcing fibers is non-uniform along the thickness of the sinks. This approach is typically applied in the class of granular composites, also known as Functionally Graded (FG) materials, to characterize the gradual variation of isotropic constituents [74,75,76,77,78,79,80]. On the other hand, in this research the graded constituent is represented by the orthotropic reinforcing fibers, which can be also arbitrarily oriented in the planar direction. A power-law function is used to characterize the through-the-thickness variation of their volume fraction. FG materials are typically used as constituents in plates and shells [81,82,83,84,85,86], beams [87,88], micro- and nano-structures [89,90,91].

Thus, it should be noted that the plates are characterized by noticeable differences in terms of mechanical properties at the layer interfaces. For this reason, a suitable structural model must be introduced to describe accurately their mechanical behavior. As clearly highlighted in the papers by Carrera [92,93,94,95,96], laminated and sandwich structures are characterized by a piece-wise continuous displacement field along the thickness direction. The differences in terms of transverse deformability among the layers give rise to a change in the slope of the displacement components between two adjacent layers. This outcome is known as zig-zag effect. As specified in [97,98,99], this effect is well-captured by Layer-Wise (LW) models, in which the degrees of freedom are assumed as independent parameters along each layer. Nevertheless, this approach is quite onerous in terms of computational resources. Effective and accurate solutions can be obtained more easily by introducing the Murakami’s function in the displacement fields of Equivalent Single Layers (ESL) models, in which all the degrees of freedom are defined within the reference surface of the structure independently from the number of layers. The Murakami’s function, in fact, is able to capture the zig-zag effect in an accurate manner without increasing excessively the computations [100]. In general, classical models for laminated structures, such as the well-known Reissner-Mindlin (RM) theory, are not able to take into account this effect. Nevertheless, these theories could be also enhanced by adding the Murakami’s function in their kinematic models. The results that can be obtained are accurate and the computational cost is reduced, if compared to the one that characterizes higher-order ESL or LW models [92]. Therefore, in the present paper the in-plane displacement field of the RM model is enriched by the Murakami’s function in order to capture the effective behavior of sandwich plates with damaged soft-core and non-uniform mechanical properties. It should be mentioned that further details concerning Zig-zag theories can be found in the review paper by Carrera [94].

Finally, a brief description of the research outline is presented. The geometric and mechanical characterization of the plates are illustrated after this introduction in Section 2. In particular, the multi-phase approach including the Eshelby-Mori-Tanaka scheme and the Halpin-Tsai homogenization procedure is described to provide the mechanical properties of the skin. In the same Section, the damage model is introduced for the isotropic soft-core. On the other hand, Section 3 is focused on the theoretical aspects of the Reissner-Mindlin Zig-zag (RMZ) theory. The corresponding FE model is developed, and the fundamental system of equations is deducted by means of the Hamilton’s principle [36,101]. The results of the numerical applications are presented in Section 4. Here, the effect of the Murakami’s function is discussed by means of the comparison between the RM and RMZ approaches, and the proposed model is validated as well. Then, several applications are illustrated to investigate the effects of the progressive damage, the non-uniform distribution of the fiber volume fraction, the in-plane fiber orientation, and the material properties on the natural frequencies of the structures and on their corresponding mode shapes. Finally, the matrix form of the fundamental operators required in the governing equations are defined in Appendix A.

## 2. Geometric and Mechanical Characterization

**Figure 1:**
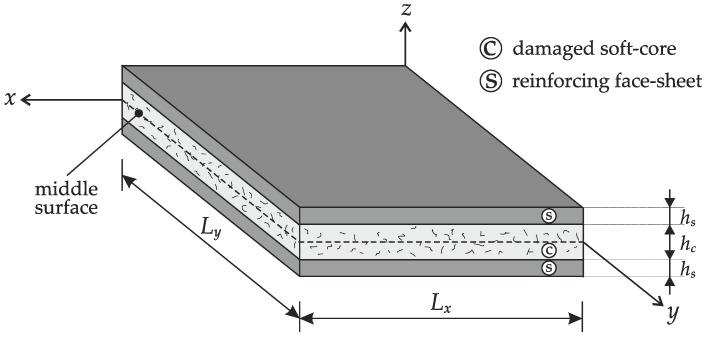
Geometric features of a laminated sandwich plate with an inner damaged soft-core.

The paper is focused on the vibrational behavior of laminated sandwich plates with an inner damaged soft-core. The plates under consideration are characterized by a planar size given by the lengths of their sides $L_{x},L_{y}$, in which x,y denote the principal directions of the local reference system. The extension along the coordinate z is specified by the overall thickness h of the composite structures, which is given by $h=2h_{s}+h_{c}$, where $h_{s}$ stands for the thickness of the external face-sheets and $h_{c}$ is the thickness of the core. Thus, the plates consist in three layers, in which the external ones are made of orthotropic materials and have the same thickness whereas the central one is isotropic. The upper and lower thickness coordinates of the generic k-th ply are denoted by $z_{k+1},z_{k}$, respectively. The geometric features of a plate element are shown in Figure 1.

The mechanical characterization of the k-th layer is carried out in terms of the engineering constants of the orthotropic material, which are the Young’s moduli $E_{11}^{\left(k\right)},E_{22}^{\left(k\right)}$, the shear moduli $G_{12}^{\left(k\right)},G_{13}^{\left(k\right)},G_{23}^{\left(k\right)}$, and the Poisson’s ratio $\nu_{12}^{\left(k\right)}$. As far as the isotropic core is concerned (k=2), only two independents parameters are needed, which are the Young’s modulus $E_{}^{\left(k\right)}=E_{11}^{\left(k\right)}=E_{22}^{\left(k\right)}$ and the Poisson’s ratio $\nu_{}^{\left(k\right)}=\nu_{12}^{\left(k\right)}$, whereas its shear modulus $G_{}^{\left(k\right)}=G_{12}^{\left(k\right)}=G_{13}^{\left(k\right)}=G_{23}^{\left(k\right)}$ is given by:

(1) $$G^{\left(k\right)}=\frac{E^{\left(k\right)}}{2\left(1+\nu^{\left(k\right)}\right)}.$$

In the following sections, the evaluation of these engineering constants is discussed for both the external layers and the soft-core. It should be specified that a perfect bonding is assumed between two adjacent layers in the proposed model.

### 2.1. Mechanical Properties of the Face-Sheets

A multiscale approach is employed to evaluate the overall mechanical properties of the three-phase composite face-sheets [38,39]. These layers are made of fiber-reinforced materials, which means that a polymer matrix (epoxy resin) is strengthened by oriented straight fibers (Carbon fibers). The third constituent is given by randomly oriented Carbon nanotubes (CNTs), which are inserted in the matrix and are used to further increase its mechanical features. The overall properties are computed by means of a two-step approach. Firstly, the Eshelby-Mori-Tanaka scheme is applied to obtain the properties of the epoxy resin including CNTs [52,53]. At this stage, the composite turns out to be isotropic due to the fact that the nanoparticles are randomly oriented, as illustrated in the paper by Shi et al. [51]. Then, the Halpin-Tsai approach is applied to combine the features of the enriched matrix with the properties of the reinforcing straight fibers [55,56,57].

At the nano-scale level, the single fiber of CNT is modeled as a linear-elastic, transversely isotropic and homogeneous cylindrical solid, as proposed in the paper by Odegard et al. [50]. Its mechanical properties are described by five parameters, which are the Hill’s elastic moduli [58,59], defined as $k_{C},l_{C},m_{C},n_{C},p_{C}$. Its density $\rho_{C}$ is required to compute the volume fraction of CNTs $V_{C}$ as follows:

(2) $$V_{C}={\left(\frac{\rho_{C}}{w_{C}\rho_{M}}-\frac{\rho_{C}}{\rho_{M}}+1\right)}^{-1},$$

where $\rho_{M}$ represents the density of the polymer matrix, whereas $w_{C}$ stands for the mass fraction of CNTs. A uniform distribution of CNTs is assumed in each layer along the thickness direction. It should be recalled that the matrix volume fraction is given by $V_{M}=1-V_{C}$. The polymer matrix is isotropic, and it is fully characterized by its Young’s modulus $E_{M}$ and Poisson’s ratio $\nu_{M}$. In the following, its bulk modulus $K_{M}$ and shear modulus $G_{M}$ are required in order to apply the Eshelby-Mori-Tanaka approach [52,53]. The following definitions are needed for this purpose:

(3) $$K_{M}=\frac{E_{M}}{3\left(1-2\nu_{M}\right)},\;G_{M}=\frac{E_{M}}{2\left(1+\nu_{M}\right)}.$$

These quantities are noticeably affected by the presence of randomly oriented CNTs. As illustrated in the paper by Shi et al. [51], if the agglomeration of CNTs is neglected, the bulk modulus $K_{M}^{*}$ and the shear modulus $G_{M}^{*}$ of the enriched matrix are given by:

(4) $$K_{M}^{*}=K_{M}+\frac{V_{C}\left(\delta_{C}-3K_{M}\alpha_{C}\right)}{3\left(V_{M}+V_{C}\alpha_{C}\right)},\;G_{M}^{*}=G_{M}+\frac{V_{C}\left(\eta_{C}-2G_{M}\beta_{C}\right)}{2\left(V_{M}+V_{C}\beta_{C}\right)},$$

where the following quantities, which can be computed by defining the Hill’s elastic moduli of CNTs, are introduced:

(5) $$\begin{array}{l} \alpha_{C}=\frac{3\left(K_{M}+G_{M}\right)+k_{C}+l_{C}}{3\left(G_{M}+k_{C}\right)},\\ \beta_{C}=\frac{1}{5}\left(\frac{4G_{M}+2k_{C}+l_{C}}{3\left(G_{M}+k_{C}\right)}+\frac{4G_{M}}{G_{M}+p_{C}}+\frac{2\left(G_{M}\left(6K_{M}+8G_{M}\right)\right)}{G_{M}\left(3K_{M}+G_{M}\right)+m_{C}\left(3K_{M}+7G_{M}\right)}\right),\\ \delta_{C}=\frac{1}{3}\left(n_{C}+2l_{C}+\frac{\left(2k_{C}+l_{C}\right)\left(3K_{M}+G_{M}-l_{C}\right)}{G_{M}+k_{C}}\right),\\ \eta_{C}=\frac{1}{5}\left(\frac{2}{3}\left(n_{C}-l_{C}\right)+\frac{8G_{M}p_{C}}{G_{M}+p_{C}}+\frac{2\left(k_{C}-l_{C}\right)\left(2G_{M}+l_{C}\right)}{3\left(G_{M}+k_{C}\right)}+\frac{8m_{C}G_{M}\left(3K_{M}+4G_{M}\right)}{3K_{M}\left(m_{C}+G_{M}\right)+G_{PM}\left(7m_{C}+G_{M}\right)}\right). \end{array}$$

Finally, the evaluation of $K_{M}^{*}$ and $G_{M}^{*}$ allows to compute the Young’s modulus $E_{M}^{*}$ and the Poisson’s ratio $\nu_{M}^{*}$ of the polymer matrix enriched by CNTs:

(6) $$E_{M}^{*}=\frac{9K_{M}^{*}G_{M}^{*}}{3K_{M}^{*}+G_{M}^{*}},\;\nu_{M}^{*}=\frac{3K_{M}^{*}-2G_{M}^{*}}{6K_{M}^{*}+2G_{M}^{*}},$$

whereas its density is given by:

(7) $$\rho_{M}^{*}=\left(\rho_{C}-\rho_{M}\right)V_{C}+\rho_{M}.$$

The mechanical properties of a single CNT fiber in terms of its Hill’s elastic moduli, as well as its density, are listed in Table 1. Such properties are valid for a single-walled Carbon nanotube with 10 as chiral index and armchair structure. Further details about the mechanical characterization of CNT can be found in [38].

In order to apply the Halpin-Tsai approach that allows to compute the overall mechanical properties of the composite given by this enriched matrix including straight Carbon fibers, the Hill’s elastic moduli of the matrix $k_{M}^{*},l_{M}^{*},m_{M}^{*},n_{M}^{*},p_{M}^{*}$ are needed. For this purpose, the following definitions are introduced:

(8) $$\begin{array}{c} k_{M}^{*}=\frac{E_{M}^{*}}{2\left(1+\nu_{M}^{*}\right)\left(1-2\nu_{M}^{*}\right)},\;l_{M}^{*}=2\nu_{M}^{*}k_{M}^{*},\;m_{M}^{*}=\left(1-2\nu_{M}^{*}\right)k_{M}^{*},\\ n_{M}^{*}=2\left(1-\nu_{M}^{*}\right)k_{M}^{*},\;p_{M}^{*}=\left(1-2\nu_{M}^{*}\right)k_{M}^{*}. \end{array}$$

Analogously, the Hill’s elastic moduli of the Carbon fibers $k_{F},l_{F},m_{F},n_{F},p_{F}$ are required. The reinforcing fibers are assumed as transversely isotropic and their mechanical properties are given by the corresponding Young’s moduli $E_{11}^{F},E_{22}^{F}$, shear modulus $G_{12}^{F}$ and Poisson’s ratios $\nu_{12}^{F},\nu_{23}^{F}$. Once these quantities are known, the Hill’s elastic moduli can be easily evaluated:

(9) $$\begin{array}{c} k_{F}=\frac{E_{22}^{F}}{2\left(1-\nu_{23}^{F}-2\nu_{21}^{F}\nu_{12}^{F}\right)},\;l_{F}=2\nu_{12}^{F}k_{F},\;m_{F}=\frac{1-\nu_{23}^{F}-2\nu_{21}^{F}\nu_{12}^{F}}{1+\nu_{23}^{F}}k_{F},\\ n_{F}=2\left(1-\nu_{23}^{F}\right)\frac{E_{11}^{F}}{E_{22}^{F}}k_{F},\;p_{F}=G_{12}^{F}, \end{array}$$

where $\nu_{21}^{F}=E_{22}^{F}\nu_{12}^{F}/E_{11}^{F}$. As shown in the previous step, the density of the fibers $\rho_{F}$ is required to compute the reference value of the corresponding volume fraction ${\tilde{V}}_{F}$, once their mass fraction $w_{F}$ is defined

(10) $${\tilde{V}}_{F}={\left(\frac{\rho_{F}}{w_{F}\rho_{M}^{*}}-\frac{\rho_{F}}{\rho_{M}^{*}}+1\right)}^{-1}.$$

Such constant quantity can be multiplied by a peculiar function $f^{\left(k\right)}\left(z\right)$ which depends on the thickness coordinate z. Consequently, the volume fraction distribution of the fibers $V_{F}$ is given by $V_{F}=V_{F}\left(z\right)={\tilde{V}}_{F}f^{\left(k\right)}\left(z\right)$ and it is clearly non-uniform along the thickness of the face-sheets.

**Figure 2:**
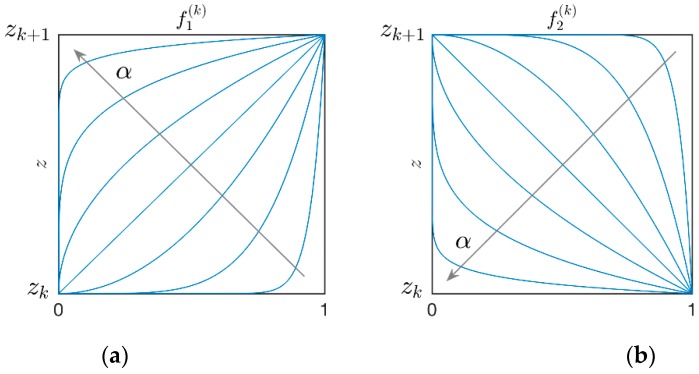
Through-the-thickness representation of the function $f^{\left(k\right)}$ employed to characterize the volume fraction distribution of the fibers in the k-th layer of the plate, for increasing values of the exponent α: (**a**) $f_{1}^{\left(k\right)}$; (**b**) $f_{2}^{\left(k\right)}$.

In the current paper, two different functions $f^{\left(k\right)}\left(z\right)$ are introduced to specify the volume fraction distribution $V_{F}$. It should be noted that they can be chosen arbitrarily in the two face-sheets of the sandwich structures under consideration. Power-law functions are used to this aim and the definitions of $f^{\left(k\right)}\left(z\right)$ are specified below:

(11) $$f^{\left(k\right)}=\left\{ \begin{array}{l} f_{1}^{\left(k\right)}\left(z\right)={\left(\frac{z-z_{k}}{z_{k+1}-z_{k}}\right)}^{\alpha }\\ f_{2}^{\left(k\right)}\left(z\right)={\left(\frac{z_{k+1}-z}{z_{k+1}-z_{k}}\right)}^{\alpha } \end{array}\right.$$

where α represents the arbitrary exponent of the distributions. As shown in Figure 2, several configurations can be obtained according to the value given to α∈[0,∞]. It should be noted that the extreme values of α, which correspond to the constant values $f^{\left(k\right)}=0,\;f^{\left(k\right)}=1$, are able to characterize a uniform distributions of the fiber along the thickness or their absence (as a consequence, the polymer matrix is the only constituent of the layer).

At this point, the Halpin-Tsai approach can be applied to obtain the Hill’s elastic moduli of the composite layers, which are denoted by k,l,m,n,p:

(12) $$\begin{array}{l} k=\frac{k_{M}^{*}\left(k_{F}+m_{M}^{*}\right)V_{M}^{*}+k_{F}\left(k_{M}^{*}+m_{M}^{*}\right)V_{F}}{\left(k_{F}+m_{M}^{*}\right)V_{M}^{*}+\left(k_{M}^{*}+m_{M}^{*}\right)V_{F}},\\ l=V_{F}l_{F}+V_{M}^{*}l_{M}^{*}+\frac{l_{F}-l_{M}^{*}}{k_{F}-k_{M}^{*}}\left(k-V_{F}k_{F}-V_{M}^{*}k_{M}^{*}\right),\\ m=m_{M}^{*}\frac{2V_{F}m_{F}\left(k_{M}^{*}+m_{M}^{*}\right)+2V_{M}^{*}m_{F}m_{M}^{*}+V_{M}^{*}k_{M}^{*}\left(m_{F}+m_{M}^{*}\right)}{2V_{F}m_{M}^{*}\left(k_{M}^{*}+m_{M}^{*}\right)+2V_{M}^{*}m_{F}m_{M}^{*}+V_{M}^{*}k_{M}^{*}\left(m_{F}+m_{M}^{*}\right)},\\ n=V_{F}n_{F}+V_{M}^{*}n_{M}^{*}+{\left(\frac{l_{F}-l_{M}^{*}}{k_{F}-k_{M}^{*}}\right)}^{2}\left(k-V_{F}k_{F}-V_{M}^{*}k_{M}^{*}\right),\\ p=\frac{\left(p_{F}+p_{M}^{*}\right)p_{M}^{*}V_{M}^{*}+2p_{F}p_{M}^{*}V_{F}}{\left(p_{F}+p_{M}^{*}\right)V_{M}^{*}+2p_{M}^{*}V_{F}}, \end{array}$$

being $V_{M}^{*}=1-V_{F}$. The engineering constants of these layers can be evaluated according to the following definitions:

(13) $$E_{11}^{\left(k\right)}=n-\frac{l^{2}}{k},\;E_{22}^{\left(k\right)}=\frac{4m\left(kn-l^{2}\right)}{kn-l^{2}+mn},\;\nu_{12}^{\left(k\right)}=\frac{l}{2k},\;G_{12}^{\left(k\right)}=G_{13}^{\left(k\right)}=p,\;G_{23}^{\left(k\right)}=m\;.$$

The introduction of the function $f^{\left(k\right)}\left(z\right)$ in the definition of $V_{F}$ causes the dependency on the thickness coordinate z of each engineering constant specified in Equation (13). Therefore, one gets $E_{11}^{\left(k\right)}\left(z\right),\;E_{22}^{\left(k\right)}\left(z\right),\;\nu_{12}^{\left(k\right)}\left(z\right),\;G_{12}^{\left(k\right)}\left(z\right),\;G_{13}^{\left(k\right)}\left(z\right)$ and $G_{23}^{\left(k\right)}\left(z\right)$, for k=1,3. Finally, the density of the composite face-sheets is given by:

(14) $$\rho^{\left(k\right)}=\left(\rho_{F}-\rho_{M}^{*}\right)V_{F}+\rho_{M}^{*}\;.$$

In the following, the same constituents are used in the external layers assuming also the same values of the mass fractions of both CNTs and fibers. The relation $\nu_{21}^{\left(k\right)}=E_{22}^{\left(k\right)}\nu_{12}^{\left(k\right)}/E_{11}^{\left(k\right)}$ is also required to compute the Poisson’s ratio $\nu_{21}^{\left(k\right)}$. As emphasized in the introduction and illustrated in the paper [38], different approaches and homogenization techniques could be used to the same aim. For instance, the rule of the mixture represents the most exploited methodology.

### 2.2. Mechanical Properties of the Damaged Matrix

The core of the sandwich structures considered in the paper is made of the same polymer matrix used in the face-sheets. Nevertheless, a damage model is introduced to provide an analytical description of an irreversible rheological process that causes the decay of the mechanical properties, in terms of engineering constants. An isotropic damage is considered in the following, which is fully characterized by a scalar D as illustrated in the book by Lemaitre and Chaboche [21]. The elastic modulus of the damaged material is given by:

(15) $$E^{\left(k\right)}=\left(1-D\right)E_{M}$$

for k=2, in which $E_{M}$ is the original value of matrix Young’s modulus, for 0≤D<1 It is clear that D=0 identifies a virgin material, whereas a fully damaged material is characterized by D=1. Having in mind relation (1), the shear modulus is subjected to the same damage. On the contrary, the Poisson’s ratio $\nu^{\left(k\right)}=\nu_{M}$ and the density $\rho^{\left(k\right)}=\rho_{M}$ of the core (k=2) are kept constant. Finally, it should be specified that the damage does not depend on the spatial coordinates and affects uniformly the core. For conciseness purposes, the mechanical properties of the undamaged epoxy resin and the Carbon fibers are summarized in Table 2.

## 3. Finite Element Model Based on A First-Order Zig-Zag Plate Theory

**Figure 3:**
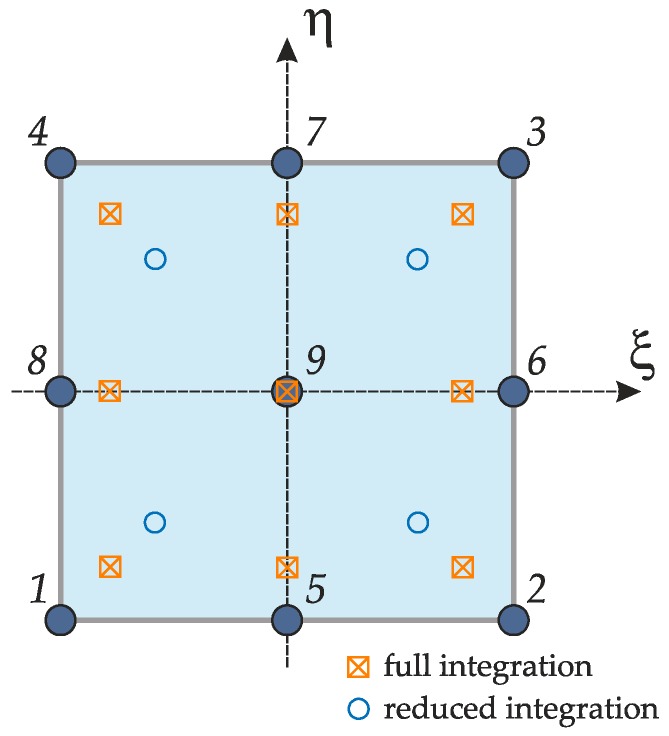
Discrete element: Node numbering and natural coordinates.

A linear theory is used to model the mechanical behavior of sandwich plates with an inner soft-core. With respect to the well-known Reissner-Mindlin (RM) theory, the in-plane expansion is enriched by two more degrees of freedom, which are able to capture the zig-zag effect [92,93,94,95,96]. Here, the corresponding (FE) formulation is presented. The displacement field for the generic e-th element is given by:

(16) $$\begin{array}{l} U_{x}^{\left(e\right)}\left(x,y,z,t\right)=u_{x}^{\left(e\right)}\left(x,y,t\right)+z\phi_{x}^{\left(e\right)}\left(x,y,t\right)+F_{z}\left(z\right)\psi_{x}^{\left(e\right)}\left(x,y,t\right)\\ U_{y}^{\left(e\right)}\left(x,y,z,t\right)=u_{y}^{\left(e\right)}\left(x,y,t\right)+z\phi_{y}^{\left(e\right)}\left(x,y,t\right)+F_{z}\left(z\right)\psi_{y}^{\left(e\right)}\left(x,y,t\right)\\ U_{z}^{\left(e\right)}\left(x,y,z,t\right)=u_{z}^{\left(e\right)}\left(x,y,t\right) \end{array}$$

in which the three-dimensional displacement components are denoted by $U_{x}^{\left(e\right)},U_{y}^{\left(e\right)},U_{z}^{\left(e\right)}$. The spatial coordinates of the plate are given by x,y,z, as shown in Figure 1, whereas t is the time variable. The zig-zag effect is modeled by means of the Murakami’s function $F_{z}\left(z\right)$ defined below for a multilayered structure:

(17) $$F_{z}={\left(-1\right)}^{k}\frac{2z}{z_{k+1}-z_{k}}-\frac{z_{k+1}+z_{k}}{z_{k+1}-z_{k}}$$

where k identifies the generic layer. This function allows to introduce a discontinuity in the slope of the three-dimensional displacements $U_{x}{}^{\left(e\right)},U_{y}{}^{\left(e\right)}$ along the thickness direction at each layer interface. Its meaning is well-described in the papers by Carrera [94]. It should be noted that the current model is characterized by seven degrees of freedom per node, two more than the classical RM approach. In particular, $u_{x}^{\left(e\right)},u_{y}^{\left(e\right)},u_{z}^{\left(e\right)}$ represent the translational displacements along x,y,z, $\phi_{x}^{\left(e\right)},\phi_{y}^{\left(e\right)}$ are the rotations about the principal axes y,x respectively, whereas $\psi_{x}^{\left(e\right)},\psi_{y}^{\left(e\right)}$ denote the magnitude of the zig-zag effect. A nine-node quadratic rectangular element is used to develop the FE formulation and each degrees of freedom is approximated by means of Lagrange interpolating functions $N_{i}^{}$, for i=1,…,9. The node numbering is illustrated in Figure 3.

Due to this approximation, the degrees of freedom can be written as a function of the corresponding nodal displacements $u_{x,i}^{\left(e\right)},u_{y,i}^{\left(e\right)},u_{z,i}^{\left(e\right)},\phi_{x,i}^{\left(e\right)},\phi_{y,i}^{\left(e\right)},\psi_{x,i}^{\left(e\right)},\psi_{y,i}^{\left(e\right)}$:

(18) $$\begin{array}{l} u_{x}^{\left(e\right)}\left(x,y,t\right)=\sum_{i=1}^{9}N_{i}^{}\left(x,y\right)u_{x,i}^{\left(e\right)}\left(t\right)=\bar{N}u_{x}^{\left(e\right)}\\ u_{y}^{\left(e\right)}\left(x,y,t\right)=\sum_{i=1}^{9}N_{i}^{}\left(x,y\right)u_{y,i}^{\left(e\right)}\left(t\right)=\bar{N}u_{y}^{\left(e\right)}\\ u_{z}^{\left(e\right)}\left(x,y,t\right)=\sum_{i=1}^{9}N_{i}^{}\left(x,y\right)u_{z,i}^{\left(e\right)}\left(t\right)=\bar{N}u_{z}^{\left(e\right)}\\ \phi_{x}^{\left(e\right)}\left(x,y,t\right)=\sum_{i=1}^{9}N_{i}^{}\left(x,y\right)\phi_{x,i}^{\left(e\right)}\left(t\right)=\bar{N}\phi_{x}^{\left(e\right)}\\ \phi_{y}^{\left(e\right)}\left(x,y,t\right)=\sum_{i=1}^{9}N_{i}^{}\left(x,y\right)\phi_{y,i}^{\left(e\right)}\left(t\right)=\bar{N}\phi_{y}^{\left(e\right)}\\ \psi_{x}^{\left(e\right)}\left(x,y,t\right)=\sum_{i=1}^{9}N_{i}^{}\left(x,y\right)\psi_{x,i}^{\left(e\right)}\left(t\right)=\bar{N}\psi_{x}^{\left(e\right)}\\ \psi_{y}^{\left(e\right)}\left(x,y,t\right)=\sum_{i=1}^{9}N_{i}^{}\left(x,y\right)\psi_{y,i}^{\left(e\right)}\left(t\right)=\bar{N}\psi_{x}^{\left(e\right)} \end{array}$$

where $\bar{N}=\left[\begin{array}{ccc} N_{1} & \cdots & N_{9} \end{array}\right]$ is the vector of the Lagrange interpolating functions. Analogously, the nodal displacements are defined in vector form as follows:

(19) $$\begin{array}{c} u_{x}^{\left(e\right)}={\left[\begin{array}{ccc} u_{x,1}^{\left(e\right)} & \cdots & u_{x,9}^{\left(e\right)} \end{array}\right]}^{T},\;u_{y}^{\left(e\right)}={\left[\begin{array}{ccc} u_{y,1}^{\left(e\right)} & \cdots & u_{y,9}^{\left(e\right)} \end{array}\right]}^{T},\;u_{z}^{\left(e\right)}={\left[\begin{array}{ccc} u_{z,1}^{\left(e\right)} & \cdots & u_{z,9}^{\left(e\right)} \end{array}\right]}^{T},\\ \phi_{x}^{\left(e\right)}={\left[\begin{array}{ccc} \phi_{x,1}^{\left(e\right)} & \cdots & \phi_{x,9}^{\left(e\right)} \end{array}\right]}^{T},\;\phi_{y}^{\left(e\right)}={\left[\begin{array}{ccc} \phi_{y,1}^{\left(e\right)} & \cdots & \phi_{y,9}^{\left(e\right)} \end{array}\right]}^{T},\\ \psi_{x}^{\left(e\right)}={\left[\begin{array}{ccc} \psi_{x,1}^{\left(e\right)} & \cdots & \psi_{x,9}^{\left(e\right)} \end{array}\right]}^{T},\;\psi_{x}^{\left(e\right)}={\left[\begin{array}{ccc} \psi_{y,1}^{\left(e\right)} & \cdots & \psi_{y,9}^{\left(e\right)} \end{array}\right]}^{T}. \end{array}$$

This notation is useful to define the vector ${\bar{u}}^{\left(e\right)}$ which includes all the nodal degrees of freedom:

(20) $${\bar{u}}^{\left(e\right)}={\left[\begin{array}{ccccccc} u_{x}^{\left(e\right)} & u_{y}^{\left(e\right)} & u_{z}^{\left(e\right)} & \phi_{x}^{\left(e\right)} & \phi_{y}^{\left(e\right)} & \psi_{x}^{\left(e\right)} & \psi_{y}^{\left(e\right)} \end{array}\right]}^{T}$$

The interpolating functions $N_{i}^{}$ assume the well-known definitions presented in the book by Reddy [11]. These polynomials are conveniently expressed as functions of the natural coordinates ξ,η, with ξ,η∈[−1,1], introduced in the so-called master element (or parent element) depicted in Figure 3. Thus, the interpolating functions become $N_{i}^{}=N_{i}^{}\left(\xi ,\eta \right)$.

The Lagrange functions $N_{i}^{}$ are also employed to define the geometric shape of each element. According to the principles of an isoparametric formulation, the coordinates $x^{\left(e\right)},y^{\left(e\right)}$ within the generic e-th element can be defined as follows:

(21) $$x^{\left(e\right)}=\sum_{i=1}^{9}N_{i}^{}\left(\xi ,\eta \right)x_{i}^{\left(e\right)},\text{​​​ }y^{\left(e\right)}=\sum_{i=1}^{9}N_{i}^{}\left(\xi ,\eta \right)y_{i}^{\left(e\right)}$$

in which the i-th node of the element under consideration is identified by the couple of nodal coordinates $x_{i}^{\left(e\right)},y_{i}^{\left(e\right)}$. Such coordinates are included in the corresponding vectors $x^{\left(e\right)},y^{\left(e\right)}$, which assume the following aspects:

(22) $$x^{e}={\left[\begin{array}{ccc} x_{1}^{\left(e\right)} & \cdots & x_{9}^{\left(e\right)} \end{array}\right]}^{T},\;y^{e}={\left[\begin{array}{ccc} y_{1}^{\left(e\right)} & \cdots & y_{9}^{\left(e\right)} \end{array}\right]}^{T}.$$

The isoparametric formulation allows to move easily all the computations in the parent space. To this aim, the Jacobian matrix J is required to perform the coordinate change. In order to define this matrix, the derivates of the interpolating functions with respect to the natural coordinates ξ,η are needed and are collected in the corresponding vectors $B_{\xi }^{},B_{\eta }^{}$ defined below:

(23) $$B_{\xi }^{}=\left[\begin{array}{ccc} \frac{\partial N_{1}}{\partial \xi } & \cdots & \frac{N_{9}}{\partial \xi } \end{array}\right],\;B_{\xi }^{}=\left[\begin{array}{ccc} \frac{\partial N_{1}}{\partial \eta } & \cdots & \frac{N_{9}}{\partial \eta } \end{array}\right].$$

At this point, the Jacobian matrix J can be introduced as specified in [11]:

(24) $$J=\left[\begin{array}{cc} \frac{\partial x^{\left(e\right)}}{\partial \xi } & \frac{\partial y^{\left(e\right)}}{\partial \xi }\\ \frac{\partial x^{\left(e\right)}}{\partial \eta } & \frac{\partial y^{\left(e\right)}}{\partial \eta } \end{array}\right]=\left[\begin{array}{cc} \sum_{i=1}^{9}x_{i}^{\left(e\right)}\frac{\partial N_{i}^{}}{\partial \xi } & \sum_{i=1}^{9}y_{i}^{\left(e\right)}\frac{\partial N_{i}^{}}{\partial \xi }\\ \sum_{i=1}^{9}x_{i}^{\left(e\right)}\frac{\partial N_{i}^{}}{\partial \eta } & \sum_{i=1}^{9}y_{i}^{\left(e\right)}\frac{\partial N_{i}^{}}{\partial \eta } \end{array}\right]=\left[\begin{array}{cc} B_{\xi }^{}x^{\left(e\right)} & B_{\xi }^{}y^{\left(e\right)}\\ B_{\eta }^{}x^{\left(e\right)} & B_{\eta }^{}y^{\left(e\right)} \end{array}\right].$$

Assuming that the determinant of the Jacobian matrix is positive, the matrix J can be inverted. Since in the following only regular rectangular elements are considered, this assumption is always satisfied and the matrix $J^{-1}$ is admissible and can be used to compute the derivatives of the interpolating functions in the physical domain defined by the coordinates x,y. The following relation is needed for this purpose:

(25) $$\left[\begin{array}{c} B_{x}^{}\\ B_{y}^{} \end{array}\right]=J^{-1}\left[\begin{array}{c} B_{\xi }^{}\\ B_{\eta }^{} \end{array}\right]$$

where $B_{x}^{}$ and $B_{x}^{}$ collect the derivatives of the shape functions with respect to x and y, respectively. These operators are required to define the compatibility equations of the RMZ model. In particular, the three-dimensional strain components for a rectangular plate can be obtained by means of the elasticity equations applied to the displacement fields (16) and assume the following definitions for the e-th element:

(26) $$\begin{array}{l} \varepsilon_{xx}^{\left(e\right)}=\frac{\partial U_{x}^{\left(e\right)}}{\partial x}=\frac{\partial u_{x}^{\left(e\right)}}{\partial x}+z\frac{\partial \phi_{x}^{\left(e\right)}}{\partial x}+F_{z}\frac{\partial \psi_{x}^{\left(e\right)}}{\partial x}=\varepsilon_{x}^{0\left(e\right)}+zk_{x}^{0\left(e\right)}+F_{z}\varepsilon_{x}^{1}=B_{x}^{}u_{x}^{\left(e\right)}+zB_{x}^{}\phi_{x}^{\left(e\right)}+F_{z}B_{x}^{}\psi_{x}^{\left(e\right)}\\ \varepsilon_{yy}^{\left(e\right)}=\frac{\partial U_{y}^{\left(e\right)}}{\partial y}=\frac{\partial u_{y}^{\left(e\right)}}{\partial y}+z\frac{\partial \phi_{y}^{\left(e\right)}}{\partial y}+F_{z}\frac{\partial \psi_{y}^{\left(e\right)}}{\partial y}=\varepsilon_{y}^{0\left(e\right)}+zk_{y}^{0\left(e\right)}+F_{z}\varepsilon_{y}^{1}=B_{y}^{}u_{y}^{\left(e\right)}+zB_{y}^{}\phi_{y}^{\left(e\right)}+F_{z}B_{y}^{}\psi_{y}^{\left(e\right)}\\ \gamma_{xy}^{\left(e\right)}=\frac{\partial U_{y}^{\left(e\right)}}{\partial x}+\frac{\partial U_{x}^{\left(e\right)}}{\partial y}=\frac{\partial u_{y}^{\left(e\right)}}{\partial x}+\frac{\partial u_{x}^{\left(e\right)}}{\partial y}+z\left(\frac{\partial \phi_{y}^{\left(e\right)}}{\partial x}+\frac{\partial \phi_{x}^{\left(e\right)}}{\partial y}\right)+F_{z}\left(\frac{\partial \psi_{y}^{\left(e\right)}}{\partial x}+\frac{\partial \psi_{x}^{\left(e\right)}}{\partial y}\right)=\\ \;=\gamma_{xy}^{0\left(e\right)}+zk_{xy}^{0\left(e\right)}+F_{z}\gamma_{xy}^{1\left(e\right)}=B_{x}^{}u_{y}^{\left(e\right)}+B_{y}^{}u_{x}^{\left(e\right)}+z\left(B_{x}^{}\phi_{y}^{\left(e\right)}+B_{y}^{}\phi_{x}^{\left(e\right)}\right)+F_{z}\left(B_{x}^{}\psi_{y}^{\left(e\right)}+B_{y}^{}\psi_{x}^{\left(e\right)}\right)\\ \gamma_{xz}^{\left(e\right)}=\frac{\partial U_{z}^{\left(e\right)}}{\partial x}+\frac{\partial U_{x}^{\left(e\right)}}{\partial z}=\frac{\partial u_{z}^{\left(e\right)}}{\partial x}+\phi_{x}^{\left(e\right)}+\frac{\partial F_{z}}{\partial z}\psi_{x}^{\left(e\right)}=\gamma_{xz}^{0\left(e\right)}+\frac{\partial F_{z}}{\partial z}\gamma_{xz}^{1\left(e\right)}=B_{x}^{}u_{z}^{\left(e\right)}+\bar{N}\phi_{x}^{\left(e\right)}+\frac{\partial F_{z}}{\partial z}\bar{N}\psi_{x}^{\left(e\right)}\\ \gamma_{yz}^{\left(e\right)}=\frac{\partial U_{z}^{\left(e\right)}}{\partial y}+\frac{\partial U_{y}^{\left(e\right)}}{\partial z}=\frac{\partial u_{z}^{\left(e\right)}}{\partial y}+\phi_{y}^{\left(e\right)}+\frac{\partial F_{z}}{\partial z}\psi_{y}^{\left(e\right)}=\gamma_{yz}^{0\left(e\right)}+\frac{\partial F_{z}}{\partial z}\gamma_{yz}^{1\left(e\right)}=B_{y}^{}u_{z}^{\left(e\right)}+\bar{N}\phi_{y}^{\left(e\right)}+\frac{\partial F_{z}}{\partial z}\bar{N}\psi_{y}^{\left(e\right)}, \end{array}$$

where $\varepsilon_{xx}^{\left(e\right)},\varepsilon_{yy}^{\left(e\right)},\gamma_{xy}^{\left(e\right)}$ are the membrane strains, and $\gamma_{xz}^{\left(e\right)},\gamma_{yz}^{\left(e\right)}$ are the transverse shear strains. It should be observed that the normal strain along the thickness direction $\varepsilon_{zz}^{\left(e\right)}$ is omitted since the plane-strain assumption entails that $\varepsilon_{zz}^{\left(e\right)}=0$. The generalized strains related to the plate middle surface can be easily defined from relations (26). The superscript “0” denotes those quantities that are included also in the well-known RM theory, whereas the terms related to the zig-zag effect are identified by the superscript “1”. In particular, it should be noted that $\varepsilon_{x}^{0\left(e\right)},\varepsilon_{y}^{0\left(e\right)},\gamma_{xy}^{0\left(e\right)}$ are the well-known membrane strains, $k_{x}^{0\left(e\right)},k_{y}^{0\left(e\right)},k_{xy}^{0\left(e\right)}$ the bending and twisting curvatures, and $\gamma_{xz}^{0\left(e\right)},\gamma_{yz}^{0\left(e\right)}$ the shear strains, which are also defined in the RM theory [36].

The constitutive equations are now used to characterize the stress components in the k-th layer of the laminate. The following definitions imply that the plane-stress assumption $\sigma_{zz}^{k\left(e\right)}=0$ is assumed by hypothesis, whereas the other stress components are given by:

(27) $$\begin{array}{l} \sigma_{xx}^{k\left(e\right)}={\bar{Q}}_{11}^{\left(k\right)}\varepsilon_{xx}^{\left(e\right)}+{\bar{Q}}_{12}^{\left(k\right)}\varepsilon_{yy}^{\left(e\right)}+{\bar{Q}}_{16}^{\left(k\right)}\gamma_{xy}^{\left(e\right)}\\ \sigma_{yy}^{k\left(e\right)}={\bar{Q}}_{12}^{\left(k\right)}\varepsilon_{xx}^{\left(e\right)}+{\bar{Q}}_{22}^{\left(k\right)}\varepsilon_{yy}^{\left(e\right)}+{\bar{Q}}_{26}^{\left(k\right)}\gamma_{xy}^{\left(e\right)}\\ \tau_{xy}^{k\left(e\right)}={\bar{Q}}_{16}^{\left(k\right)}\varepsilon_{xx}^{\left(e\right)}+{\bar{Q}}_{26}^{\left(k\right)}\varepsilon_{yy}^{\left(e\right)}+{\bar{Q}}_{66}^{\left(k\right)}\gamma_{xy}^{\left(e\right)}\\ \tau_{xz}^{k\left(e\right)}={\bar{Q}}_{44}^{\left(k\right)}\gamma_{xz}^{\left(e\right)}+{\bar{Q}}_{45}^{\left(k\right)}\gamma_{yz}^{\left(e\right)}\\ \tau_{yz}^{k\left(e\right)}={\bar{Q}}_{45}^{\left(k\right)}\gamma_{xz}^{\left(e\right)}+{\bar{Q}}_{55}^{\left(k\right)}\gamma_{yz}^{\left(e\right)} \end{array}$$

in which ${\bar{Q}}_{ij}^{\left(k\right)}$ denotes the stiffnesses of the k-th orthotropic layer evaluated in the geometric reference system. These parameters have the same meaning in each finite element, since the mechanical properties of the structure do not vary in the plate middle surface. Their well-known definition, which depends on the parameters $Q_{ij}^{\left(k\right)}$ expressed as a function of the engineering constants of the k-layer defined below, can be found in the book by Reddy [36]:

(28) $$Q_{11}^{\left(k\right)}=\frac{E_{11}^{\left(k\right)}}{1-\nu_{12}^{\left(k\right)}\nu_{21}^{\left(k\right)}},\;Q_{22}^{\left(k\right)}=\frac{E_{22}^{\left(k\right)}}{1-\nu_{12}^{\left(k\right)}\nu_{21}^{\left(k\right)}},\;Q_{12}^{\left(k\right)}=\frac{\nu_{12}^{\left(k\right)}E_{22}^{\left(k\right)}}{1-\nu_{12}^{\left(k\right)}\nu_{21}^{\left(k\right)}},\;Q_{66}^{\left(k\right)}=G_{12}^{\left(k\right)},\;Q_{44}^{\left(k\right)}=G_{13}^{\left(k\right)},\;Q_{55}^{\left(k\right)}=G_{23}^{\left(k\right)}\;.$$

Quantities in (28) for k=1,3 depend on the thickness coordinate z, since the reinforcing fibers of the face-sheets are characterized by a non-uniform distribution in this direction. Each orthotropic layer can be also characterized by an arbitrary orientation $\theta^{\left(k\right)}$. The notation $\left(\theta^{\left(1\right)}/\mathrm{core}/\theta^{\left(3\right)}\right)$ is used in the next Sections to specify the orientations of the reinforcing fibers in the face-sheets and the consequent lamination scheme. Conventionally, in the above notation the layer numbering always starts from the bottom surface of the plate.

At this point, the Hamilton’s variational principle should be applied to obtain the governing equations for the dynamic problem under consideration [36,101]. If $t_{1},t_{2}$ specify the boundary values of the considered time interval, the variational principle assumes the following aspect within the e-th discrete element:

(29) $$\int_{t_{1}}^{t_{2}}\left(\delta {Κ}^{\left(e\right)}-\delta \Phi^{\left(e\right)}\right)dt=0$$

where $\delta {Κ}^{\left(e\right)}$ is the variation of the kinetic energy, whereas $\delta \Phi^{\left(e\right)}$ represents the variation of the elastic strain energy. The kinetic energy of the sandwich structure $\delta {Κ}^{\left(e\right)}$ is given by:

(30) $$\delta {Κ}^{\left(e\right)}=-\sum_{k=1}^{3}\int_{x}^{}\int_{y}^{}\int_{z_{k}}^{z_{k+1}}\rho^{\left(k\right)}\left(\delta U_{x}^{\left(e\right)}{\ddot{U}}_{x}^{\left(e\right)}+\delta U_{y}^{\left(e\right)}{\ddot{U}}_{y}^{\left(e\right)}+\delta U_{y}^{\left(e\right)}{\ddot{U}}_{y}^{\left(e\right)}\right)dxdydz$$

where the double-dot notation specifies the second-order derivatives with respect to the time variable. On the other hand, the elastic strain energy $\delta \Phi^{\left(e\right)}$ is defined as follows:

(31) $$\delta \Phi^{\left(e\right)}=\sum_{k=1}^{3}\int_{x}^{}\int_{y}^{}\int_{z_{k}}^{z_{k+1}}\left(\sigma_{xx}^{k\left(e\right)}\delta \varepsilon_{xx}^{\left(e\right)}+\sigma_{yy}^{k\left(e\right)}\delta \varepsilon_{yy}^{\left(e\right)}+\tau_{xy}^{k\left(e\right)}\delta \gamma_{xy}^{\left(e\right)}+\tau_{xz}^{k\left(e\right)}\delta \gamma_{xz}^{\left(e\right)}+\tau_{yz}^{k\left(e\right)}\delta \gamma_{yz}^{\left(e\right)}\right)dxdydz$$

The proper mathematical manipulations of the elastic strain energy provide the definitions of the stress resultants as the through-the-thickness integrals of the stress components. The stress resultants can be written in matrix form by means of the following relations, which provide their definitions as a function of the generalized strain components introduced before:

(32) $$\left[\begin{array}{c} N_{x}^{0\left(e\right)}\\ N_{y}^{0\left(e\right)}\\ N_{xy}^{0\left(e\right)}\\ M_{x}^{0\left(e\right)}\\ M_{y}^{0\left(e\right)}\\ M_{xy}^{0\left(e\right)}\\ T_{x}^{0\left(e\right)}\\ T_{y}^{0\left(e\right)}\\ N_{x}^{1\left(e\right)}\\ N_{y}^{1\left(e\right)}\\ N_{xy}^{1\left(e\right)}\\ T_{x}^{1\left(e\right)}\\ T_{y}^{1\left(e\right)} \end{array}\right]=\left[\begin{array}{ccccccccccccc} A_{11} & A_{12} & A_{16} & B_{11} & B_{12} & B_{16} & 0 & 0 & F_{11} & F_{12} & F_{16} & 0 & 0\\ A_{12} & A_{22} & A_{26} & B_{12} & B_{22} & B_{26} & 0 & 0 & F_{12} & F_{22} & F_{26} & 0 & 0\\ A_{16} & A_{26} & A_{66} & B_{16} & B_{26} & B_{66} & 0 & 0 & F_{16} & F_{26} & F_{66} & 0 & 0\\ B_{11} & B_{12} & B_{16} & D_{11} & D_{12} & D_{16} & 0 & 0 & G_{11} & G_{12} & G_{16} & 0 & 0\\ B_{12} & B_{22} & B_{26} & D_{12} & D_{22} & D_{26} & 0 & 0 & G_{12} & G_{22} & G_{26} & 0 & 0\\ B_{16} & B_{26} & B_{66} & D_{16} & D_{26} & D_{66} & 0 & 0 & G_{16} & G_{26} & G_{66} & 0 & 0\\ 0 & 0 & 0 & 0 & 0 & 0 & \kappa A_{44} & \kappa A_{45} & 0 & 0 & 0 & \kappa H_{44} & \kappa H_{45}\\ 0 & 0 & 0 & 0 & 0 & 0 & \kappa A_{45} & \kappa A_{55} & 0 & 0 & 0 & \kappa H_{45} & \kappa H_{55}\\ F_{11} & F_{12} & F_{16} & G_{11} & G_{12} & G_{16} & 0 & 0 & L_{11} & L_{12} & L_{16} & 0 & 0\\ F_{12} & F_{22} & F_{26} & G_{12} & G_{22} & G_{26} & 0 & 0 & L_{12} & L_{22} & L_{26} & 0 & 0\\ F_{16} & F_{26} & F_{66} & G_{16} & G_{26} & G_{66} & 0 & 0 & L_{16} & L_{26} & L_{66} & 0 & 0\\ 0 & 0 & 0 & 0 & 0 & 0 & \kappa H_{44} & \kappa H_{45} & 0 & 0 & 0 & \kappa P_{44} & \kappa P_{45}\\ 0 & 0 & 0 & 0 & 0 & 0 & \kappa H_{45} & \kappa H_{55} & 0 & 0 & 0 & \kappa P_{45} & \kappa P_{55} \end{array}\right]\left[\begin{array}{c} \varepsilon_{x}^{0\left(e\right)}\\ \varepsilon_{y}^{0\left(e\right)}\\ \gamma_{xy}^{0\left(e\right)}\\ k_{x}^{0\left(e\right)}\\ k_{y}^{0\left(e\right)}\\ k_{xy}^{0\left(e\right)}\\ \gamma_{xz}^{0\left(e\right)}\\ \gamma_{yz}^{0\left(e\right)}\\ \varepsilon_{x}^{1\left(e\right)}\\ \varepsilon_{y}^{1\left(e\right)}\\ \gamma_{xy}^{1\left(e\right)}\\ \gamma_{xz}^{1\left(e\right)}\\ \gamma_{yz}^{1\left(e\right)} \end{array}\right]\;.$$

The superscripts “0” and “1” have the same meaning discussed previously. In particular, $N_{x}^{0\left(e\right)},N_{y}^{0\left(e\right)},N_{xy}^{0\left(e\right)}$ are the membrane forces, $M_{x}^{0\left(e\right)},M_{y}^{0\left(e\right)},M_{xy}^{0\left(e\right)}$ the bending and twisting moments, and $T_{x}^{0\left(e\right)},T_{y}^{0\left(e\right)}$ the shear forces, which are included also in the RM theory [11]. The other terms are related to the zig-zag effect. The elements of the constitutive operator in (32) are now discussed. Firstly, the following terms appear also in the RM model and have the same meaning [11]:

(33) $$A_{ij}=\sum_{k=1}^{3}\int_{z_{k}}^{z_{k+1}}{\bar{Q}}_{ij}^{\left(k\right)}dz,\;B_{ij}=\sum_{k=1}^{3}\int_{z_{k}}^{z_{k+1}}z{\bar{Q}}_{ij}^{\left(k\right)}dz,\;D_{ij}=\sum_{k=1}^{3}\int_{z_{k}}^{z_{k+1}}z^{2}{\bar{Q}}_{ij}^{\left(k\right)}dz.$$

On the other hand, the stress resultants related to the zig-zag effect require the following definitions, which include the Murakami’s function and its derivative with respect to the thickness coordinate:

(34) $$\begin{array}{c} F_{ij}=\sum_{k=1}^{3}\int_{z_{k}}^{z_{k+1}}F_{z}{\bar{Q}}_{ij}^{\left(k\right)}dz,\;G_{ij}=\sum_{k=1}^{3}\int_{z_{k}}^{z_{k+1}}zF_{z}{\bar{Q}}_{ij}^{\left(k\right)}dz,\;H_{ij}=\sum_{k=1}^{3}\int_{z_{k}}^{z_{k+1}}\frac{\partial F_{z}}{\partial z}{\bar{Q}}_{ij}^{\left(k\right)}dz,\\ L_{ij}=\sum_{k=1}^{3}\int_{z_{k}}^{z_{k+1}}F_{z}^{2}{\bar{Q}}_{ij}^{\left(k\right)}dz,\;P_{ij}=\sum_{k=1}^{3}\int_{z_{k}}^{z_{k+1}}{\left(\frac{\partial F_{z}}{\partial z}\right)}^{2}{\bar{Q}}_{ij}^{\left(k\right)}dz. \end{array}$$

It should be specified that the integrals in (33)–(34) are computed numerically since the stiffnesses ${\bar{Q}}_{ij}^{\left(k\right)}$ can be arbitrary functions of z, due to the dependency on the thickness coordinate introduced by relation (11). Finally, it is important to specify that the shear forces need the shear correction factor κ. The value of 5/6 is used to this aim.

The system of dynamic equations for the problem under consideration represents the main result of the application of the Hamilton’s principle. The fundamental equation for the generic element e is given by:

(35) $$K^{\left(e\right)}{\bar{u}}^{\left(e\right)}+M^{\left(e\right)}{\ddot{\bar{u}}}^{\left(e\right)}=0$$

where $K^{\left(e\right)}$ is the element stiffness matrix, $M^{\left(e\right)}$ the element mass matrix, and ${\ddot{\bar{u}}}^{\left(e\right)}$ the vector of the second-order time derivatives of the element degrees of freedom included in ${\bar{u}}^{\left(e\right)}$. The fundamental operators $K^{\left(e\right)}$ assumes the following definitions:

(36) $$K^{\left(e\right)}=\left[\begin{array}{cccc} K_{11} & \cdots & \cdots & K_{17}\\ \vdots & \ddots & & \vdots \\ \vdots & & \ddots & \vdots \\ K_{71} & \cdots & \cdots & K_{77} \end{array}\right]\;.$$

On the other hand, the mass matrix $M^{\left(e\right)}$ is given by:

(37) $$M^{\left(e\right)}=\left[\begin{array}{cccc} M_{11} & \cdots & \cdots & M_{17}\\ \vdots & \ddots & & \vdots \\ \vdots & & \ddots & \vdots \\ M_{71} & \cdots & \cdots & M_{77} \end{array}\right].$$

The matrices $K_{ij}$ and $M_{ij}$, for i,j=1,…,7 are defined in the Appendix A. In particular, the following inertia terms are required to compute the mass matrix:

(38) $$\begin{array}{c} I_{0}=\sum_{k=1}^{3}\int_{z_{k}}^{z_{k+1}}\rho^{\left(k\right)}dz,\;I_{1}=\sum_{k=1}^{3}\int_{z_{k}}^{z_{k+1}}z\rho^{\left(k\right)}dz,\;I_{2}=\sum_{k=1}^{3}\int_{z_{k}}^{z_{k+1}}z^{2}\rho^{\left(k\right)}dz,\\ I_{3}=\sum_{k=1}^{3}\int_{z_{k}}^{z_{k+1}}F_{z}\rho^{\left(k\right)}dz,\;I_{4}=\sum_{k=1}^{3}\int_{z_{k}}^{z_{k+1}}zF_{z}\rho^{\left(k\right)}dz,\;I_{5}=\sum_{k=1}^{3}\int_{z_{k}}^{z_{k+1}}F_{z}^{2}\rho^{\left(k\right)}dz. \end{array}$$

It can be easily observed that the terms $I_{0},I_{1},I_{2}$ are included also in the RM theory. On the other hand, the parameters $I_{3},I_{4},I_{5}$ are linked to the zig-zag effect and include the Murakami’s function. It should be specified that these integrals are computed numerically, since the density $\rho^{\left(k\right)}$, for k=1,3, is an arbitrary function of the thickness coordinate.

**Figure 4:**
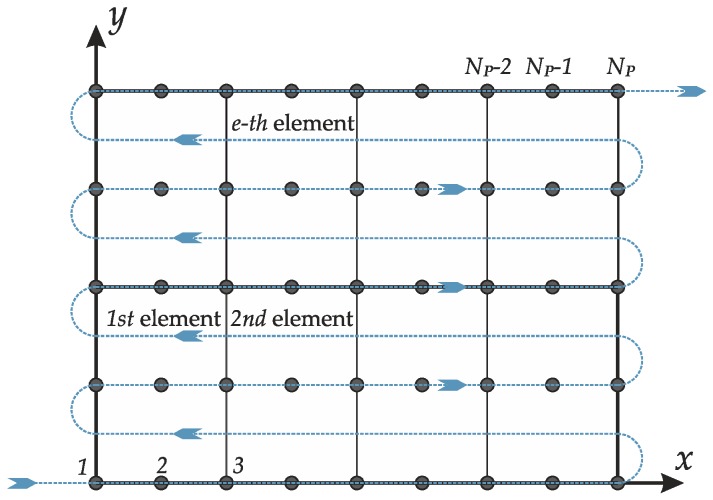
Discrete domain, identification of the elements and node numbering.

The fundamental Equation (35) is valid for each discrete subdomain at the element level. The well-known assembly procedure is required to obtain the corresponding global system of equations. This approach allows to enforce automatically the displacement continuity at the element interfaces. Therefore, a $C^{0}$ compatibility requirement is satisfied in the current approach. Once the global matrices are obtained, the fundamental system of equations assumes the following aspect:

(39) $$Ku+M\ddot{u}=0$$

in which K,M are the global stiffness and mass matrices, respectively. The degrees of freedom of the whole structure are collected in the vector u. The nodal displacements are listed following the scheme specified by the dashed line in Figure 4, where an example of a discrete plate domain is also depicted.

If $N_{P}$ denotes the number of nodes, the model is characterized by $N_{dofs}=7N_{P}$ as number of degrees of freedom. Consequently, the vector u can be written as follows:

(40) $$\begin{array}{l} u=[\begin{array}{ccc} \underset{1\to N_{P}}{u_{x,1}\;\cdots \;u_{x,N_{P}}} & \underset{N_{P}+1\to 2N_{P}}{u_{y,1}\;\cdots \;u_{y,N_{P}}} & \underset{2N_{P}+1\to 3N_{P}}{u_{z,1}\;\cdots \;u_{z,N_{P}}} \end{array}\\ \;{\begin{array}{cccc} \underset{3N_{P}+1\to 4N_{P}}{\phi_{x,1}\;\cdots \;\phi_{x,N_{P}}} & \underset{4N_{P}+1\to 5N_{P}}{\phi_{y,1}\;\cdots \;\phi_{y,N_{P}}} & \underset{5N_{P}+1\to 6N_{P}}{\psi_{x,1}\;\cdots \;\psi_{x,N_{P}}} & \underset{6N_{P}+1\to 7N_{P}}{\psi_{y,1}\;\cdots \;\psi_{y,N_{P}}} \end{array}]}^{T}\;. \end{array}$$

The second-order time derivatives of these quantities are collected in the vector $\ddot{u}$ following the same scheme. Finally, it should be recalled that the size of the fundamental operators K,M is given by $N_{dofs}\times N_{dofs}$. The discrete system can be solved once the proper boundary conditions along the edges of the domain are enforced. In the present paper, since only fully clamped plates are considered, all the nodal displacements related to the boundary edges are all equal to zero.

### 3.1. Numerical Computation of the Fundamental Matrices

The Gauss-Legendre quadrature rule is employed to compute the fundamental matrices K,M. By definition, the integral of a generic function G(x,y) defined in a two-dimensional domain can be evaluated as follows:

(41) $$\int_{x}\int_{y}G\left(x,y\right)dxdy=\int_{-1}^{1}\int_{-1}^{1}G\left(\xi ,\eta \right)\det Jd\xi d\eta$$

in which the determinant of the Jacobian matrix detJ is introduced. Therefore, the integral is computed in the parent space in which the reference system is given by natural coordinates ξ,η. From the numerical point of view, this integral can be converted into the following weighted linear sum:

(42) $$\int_{-1}^{1}\int_{-1}^{1}G\left(\xi ,\eta \right)\det Jd\xi d\eta \approx \sum_{I=1}^{M}\sum_{J=1}^{N}G\left(\xi_{I},\eta_{J}\right){\det J|}_{\xi_{I},\eta_{J}}W_{I}W_{J}$$

where $W_{I},W_{J}$ are the weighting coefficients, whereas $\xi_{I},\eta_{J}$ are the points in which the integral is computed. These nodes are the roots of Legendre polynomials [11]. The full integration is performed considering 9 evaluation points, whereas the reduced one is carried out in four points only. The position of these nodes is shown in Figure 3. It should be specified that the reduced integration is employed only to compute the elements of the stiffness matrix related to the shear forces in order to avoid the shear locking issue [11]. The analytical values of the weighting coefficients for each root of the Legendre polynomials can be found in the book by Reddy [11].

### 3.2. Evaluation of the Natural Frequencies

The free vibration analysis is based on a generalized eigenvalue problem from the analytical point of view. In particular, the following relation allows to compute the circular frequencies ω of the structures under consideration:

(43) $$\left(K-\omega^{2}M\right)d=0$$

where the modal amplitudes are collected in the vector d. Once relation (43) is solved, the natural frequencies f measured in Hz can be easily computed as f=ω/2π.

## 4. Numerical Applications

The numerical applications presented in this Section aim to evaluate the natural frequencies of several fully clamped sandwich plates. The geometric features are the same in each computation. In particular, a square domain defined by $L_{x}=L_{y}=2.5\;m$ is considered. The thickness of the external layers is $h_{s}=0.02\;m$, whereas the soft-core is defined by $h_{c}=0.06\;m$. Each structure is subdivided into 100 finite elements as far as the discrete domain is concerned.

Firstly, the validity of the current approach based on the RMZ model is proved and compared with the results that could be obtained by means of the well-known RM theory. To this aim, a three-dimensional FE model is built through a commercial code. Secondly, the model is also validated with respect to the application of non-uniform distributions of the reinforcing fibers along the plate thickness. For this purpose, the results are compared with the ones available in the literature. Then, several parametric investigations are presented to discuss the effects of the damage, the through-the-thickness distributions of the reinforcing fibers, the lamination scheme and the in-plane orientation of the fibers, the mechanical properties of fibers and CNTs on the vibrational response.

### 4.1. Influence of the Murakami’s Function and Validation of the RMZ

The first test aims to prove the need of the Murakami’s function when the mechanical behavior of sandwich structures with an inner soft-core must be analyzed. In this application, the core is made by a virgin material (D=0) and the fibers are uniformly placed along the thickness of the external face-sheets. The mechanical characterization is fully accomplished by setting $w_{C}=0.05$ and $w_{F}=0.80$. The natural frequencies are obtained by using the RM and the RMZ models, for three different lamination schemes. The same structures are investigated by means of a three-dimensional FE commercial code (twenty-node brick elements), denoted by 3D-FE in the following. The software Strand7 is employed for this purpose. The first ten natural frequencies for the sandwich plate with an inner soft-core under consideration are shown in Table 3, where the percentage differences (%diff) of the RMZ and RM solutions with respect to the 3D-FE results are also highlighted. The following aspects can be observed:

- The RMZ theory provides natural frequencies that are close to the results given by the reference solution (3D-FE). In fact, the maximum percentage difference is about 5% for higher modes. This difference is satisfactory having in mind the approximation introduced by a two-dimensional ESL theory;
- The computational cost is very different. In particular, the number of degrees of freedom in the 3D-FE model is ten times the one needed by the RMZ theory to obtain similar values;
- The RM model is not adequate to evaluate the natural frequencies of a sandwich soft-core structure, as it can be observed by the percentage differences with respect to the reference solution. The number of degrees of freedom in this circumstance is even lower if compared to the other models, but the computational saving cannot justify the poor approximation of the solution.

### 4.2. Validation of the Model with Respect to Non-Uniform Distributions of the Reinforcing Fibers

The current approach is validated also with respect to the application of non-uniform distribution of the reinforcing fibers in the thickness direction. In the paper by Lei et al. [71], a fully clamped square plate, characterized by the aspect ratio $L_{x}/h=10$, is analyzed by means of the kp-Ritz method. The structure is made of an epoxy resin ($E_{M}=2.1\;\mathrm{GPa}$, $\nu_{M}=0.34$, $\rho_{M}=1150\;\mathrm{kg}/m^{3}$) reinforced by aligned fibers of CNTs. This configuration can be modeled as a two-phase composite, in which the Carbon fibers are characterized by the following mechanical properties $E_{11}^{F}=5.6466\;\mathrm{TPa}$, $E_{22}^{F}=7.0800\;\mathrm{TPa}$, $G_{12}^{F}=1.9445\;\mathrm{TPa}$, $\nu_{12}^{F}=0.175$, $\rho_{F}=1400\;\mathrm{kg}/m^{3}$. It should be noted that the approach presented in this paper can be used to evaluate the overall mechanical properties of this structure by neglecting the effect of the randomly oriented CNT particles scattered in the matrix. In other words, one gets $E_{M}^{*}=E_{M}^{}$, $\nu_{M}^{*}=\nu_{M}^{}$ and $\rho_{M}^{*}=\rho_{M}^{}$. On the other hand, the aligned CNTs assume the same role of the straight fibers. Equation (10) is employed to obtain the value of $w_{F}$ which provides the constant ${\tilde{V}}_{F}=0.11$ specified in the reference paper.

In order to validate the current methodology, the plate is made of two orthotropic layers of equal thickness (no soft-core is included), characterized by non-uniform distributions of the CNT fibers. “Case 1” is obtained by $f_{1}^{\left(1\right)},f_{2}^{\left(2\right)}$ with α=1, whereas “Case 2” is given by $f_{2}^{\left(1\right)},f_{1}^{\left(2\right)}$ with α=1. The frequencies are presented in Table 4 in dimensionless form as

(44) $$\varpi =\omega \frac{L_{x}^{2}}{h}\sqrt{\frac{\rho_{M}}{E_{M}}}$$

in which ω denotes the circular frequencies. As specified in the previous sections, the Halpin-Tsai (HT) model is applied for the mechanical characterization of the structure. Nevertheless, the reference solutions are obtained by means of the rule of the mixture (MIX). The same approach has been used in the paper by Bacciocchi and Tarantino [39] for similar purposes. Therefore, only in the next application these two models (MIX and HT) are considered for the sake of comparison. Small differences are obtained depending on the homogenization procedure used in the computation.

It should be emphasized that the current application does not aim to investigate the effect of the homogenization method but only the comparison with the reference solution of the frequency parameter. As it can be noted from the results shown in Table 4, a good agreement is observed with the reference solution, especially if the rule of the mixture is employed as it could be expected. In the same table, the FE results provided by a commercial code are also presented. Finally, it should be specified that the RMZ theory is considered as far as the theoretical model of the present solutions is concerned.

### 4.3. Effect of Damage

**Figure 5:**
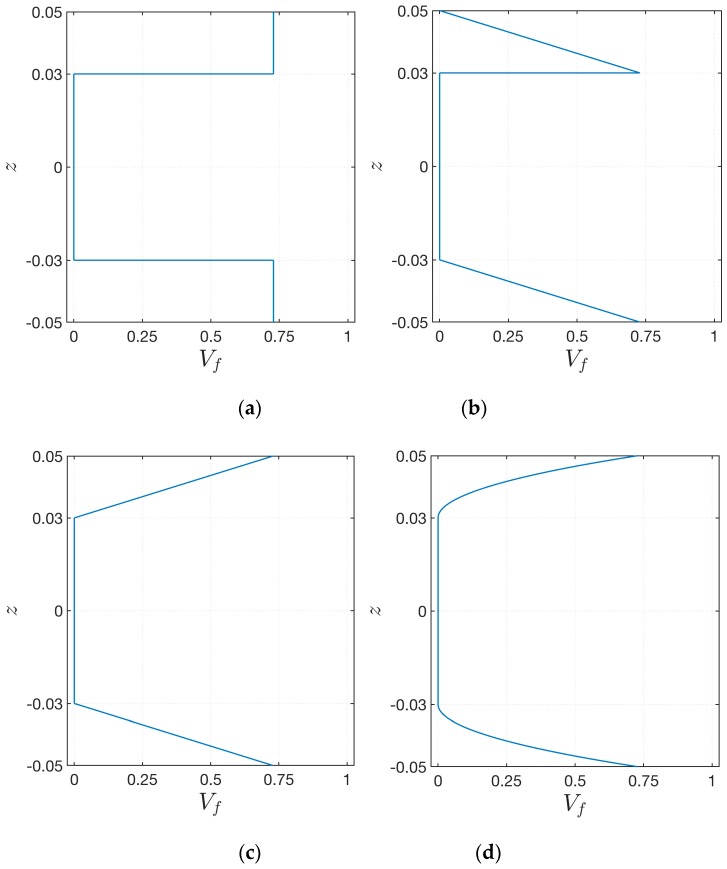
Through-the-thickness representation of the volume fraction distribution $V_{F}$ for several schemes: (**a**) Scheme 1; (**b**) Scheme 2; (**c**) Scheme 3; (**d**) Scheme 4.

The same geometric and mechanical configurations are analyzed also in this paragraph. Nevertheless, the soft-core of the structures is affected by a decay of the mechanical properties and the effect of an increasing damage parameter D∈[0,1] on the natural frequencies is discussed. For each lamination scheme, four different through-the-thickness distributions of the reinforcing fibers in the face-sheets are investigated, including the uniform one. “Scheme 1” denotes the uniform distribution of the fibers; “Scheme 2” is obtained by setting $f_{2}^{\left(1\right)}=f_{2}^{\left(3\right)}$ with α=1; “Scheme 3” is accomplished by using $f_{2}^{\left(1\right)},f_{1}^{\left(3\right)}$ with α=1; finally, “Scheme 4” has the same functions of the previous one, assuming α=2. These configurations are graphically depicted in Figure 5. The volume fraction distribution of the fibers in the core is clearly equal to zero.

**Figure 6:**
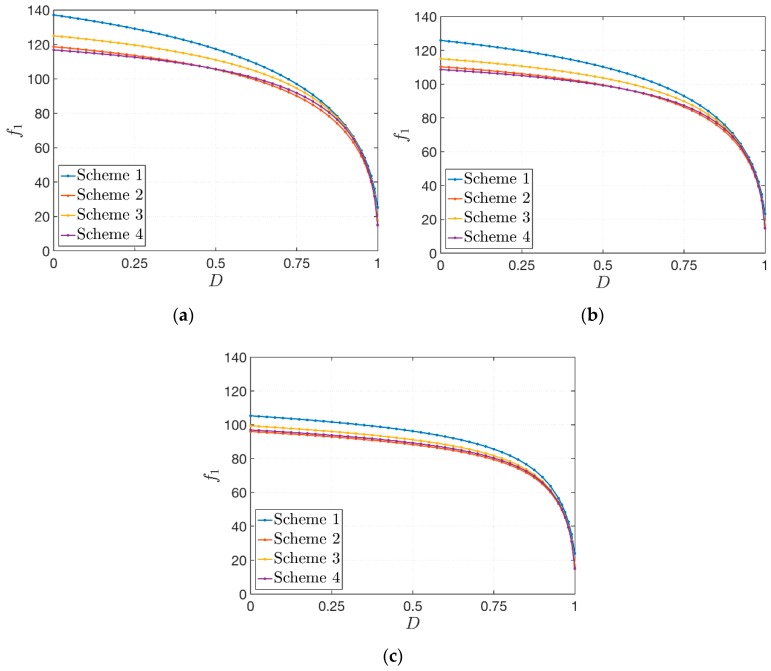
Variation of the first natural frequency $f_{1}$ [Hz] for sandwich plates with a damaged soft-core due to an increasing damage D, for three different lamination schemes: (**a**) (0°/core/0°); (**b**) (30° /core/45°); (**c**) (−45° /core/45°).

The results are presented in Figure 6, where it can be observed that the first natural frequency for each configuration depends noticeably on the parameter D. The following aspects should be noted, as well:

- The decrease of the frequency is clearly caused by the corresponding stiffness reduction of the structures. This expected tendency models accurately the physical behavior of structures with a lower value of stiffness. In fact, by increasing the value of D up to the unity (fully damaged core), the frequency would tend to zero;
- The same behavior is obtained for each volume fraction distribution, but the maximum value of the first frequency that can be reached depends on the through-the-thickness distributions of $V_{F}$ (Figure 5);
- These aspects can be noted for each lamination scheme. Nevertheless, depending on the in-plane fiber orientation, the value of the first frequency could change. In addition, a peculiar choice of lamination scheme could reduce the influence of the through-the-thickness distributions of the volume fraction $V_{F}$, since the curves related to the various schemes are less detached;
- Finally, similar graphs could be obtained also for higher frequencies.

### 4.4. Influence of the Exponent of the Through-the-Thickness Distribution of the Fiber Volume Fraction

The current application deals with the effect of the exponent α that characterizes the through-the-thickness distribution of $V_{F}$. For this purpose, the Scheme 2 and Scheme 3 of the previous test are considered. Nevertheless, different configurations are obtained since α∈[0,∞], as it can be seen from Figure 2.

**Figure 7:**
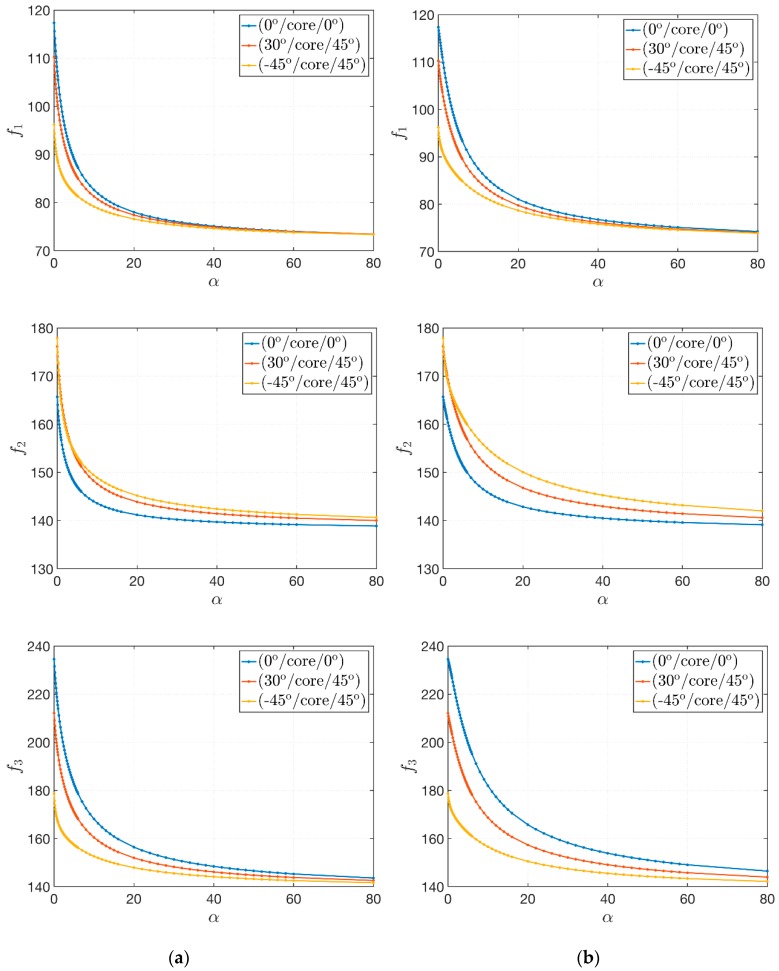
Variation of the first three natural frequencies $f_{1}$, $f_{2}$, $f_{3}$ [Hz] for sandwich plates with a damaged soft-core due to an increasing value of the exponent α of the through-the-thickness distribution of the fiber volume fraction, for two different schemes: (**a**) Scheme 2; (**b**) Scheme 3.

The geometry of the sandwich plate is kept constant and three laminations schemes are considered: (0°/core/0°), (30°/core/45°) and (−45°/core/45°). A damaged core is modeled by setting D=0.5. It should be recalled that the same values of the mass fraction of both CNTs and fibers (respectively $w_{C}=0.05$ and $w_{F}=0.80$) are employed. The proper choice of the exponent α allows to obtain also the following extreme cases. For α=0, the reinforcing fibers are uniformly distributed, and the Scheme 1 of the previous application is accomplished. On the other hand, if α tends to infinity, it is easy to verify that $V_{F}=0$ and the face-sheets are made of an undamaged polymer matrix enforced only by CNTs. In this circumstance, the stiffness of the structure reaches its minimum value. In terms of natural frequencies, the results are included between these two boundary cases. The variation of the first three natural frequencies is depicted in Figure 7. The following features can be observed:

- Similar behaviors are obtained for the three lamination schemes under investigation. For lower values of α, the corresponding curves are detached and the natural frequencies that can be obtained assume different values depending on the fiber orientation. By increasing the exponent α, the effect of the fibers decreases since $V_{F}$ draws near zero and the frequencies tends asymptotically to the same value;
- The initial choice of the through-the-thickness distribution of $V_{F}$ (Scheme 2 or Scheme 3) affects the variation of the natural frequencies. In particular, this variation is faster for Scheme 2. In fact, the slopes of the related curves are steeper, whereas the frequency variation for Scheme 3 is a little bit more gradual;
- The biggest variation of frequencies is reached for lower values of α. The decrease of the value of natural frequencies for α>20 is negligible.

### 4.5. Effect of the In-Plane Fiber Orientation

**Figure 8:**
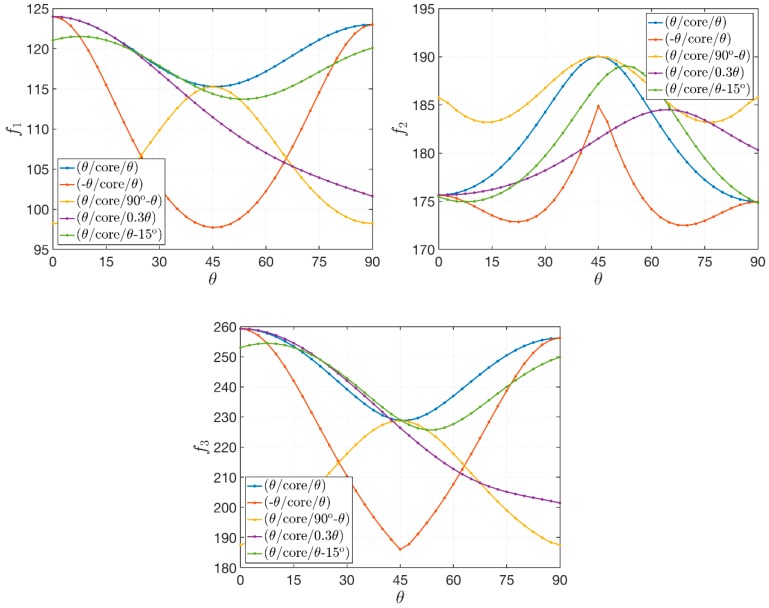
Variation of the first three natural frequencies $f_{1}$, $f_{2}$, $f_{3}$ [Hz] for a sandwich plates with a damaged soft-core for several lamination schemes depending on the angle parameter θ which defines the in-plane fiber orientation.

A damaged sandwich plate with D=0.25 is considered in the following application. The geometric features are kept constant with respect to the previous tests, whereas the engineering constants of the face-sheets can be computed assuming $w_{C}=0.05$ and $w_{F}=0.80$. A non-uniform distribution of the reinforcing fiber is defined according to the functions that describe Scheme 3, with α=0.5. The aim of this numerical application is to show the dependency of the natural frequencies on the in-plane fiber orientation. Therefore, several configurations are analyzed according to the value of an angular parameter θ. The variation of the first three natural frequencies is depicted in Figure 8 for various lamination schemes depending on θ. The graphs in Figure 8 prove the following results:

- As expected, the orientation of the fibers affects the dynamic response of the composite structures under consideration;
- If symmetric angle-ply or cross-ply laminates, as well as antisymmetric configurations, are considered, which are denoted by (θ/core/θ) and (−θ/core/θ), the extreme values of frequencies can be obtained for $\theta =0^{{}^{\circ}},45^{{}^{\circ}},90^{{}^{\circ}}$. In addition, a symmetrical behavior is obtained after reaching the value of $\theta =45^{{}^{\circ}}$;
- This regular and symmetric behavior is lost if a laminate with a general stacking sequence, such as the last two lamination schemes, is analyzed.

### 4.6. Influence of the Material Properties

Finally, this last application aims to discuss the effects of the CNT mass fraction $w_{C}$ and the fiber mass fraction $w_{F}$ on the structural response. The same geometric features are considered in this circumstance, assuming D=0.75 as damage parameters. The through-the-thickness distribution of the reinforcing fibers $V_{F}$ are defined by the functions used in Scheme 3 (Figure 5) by setting α=4. The lamination scheme is given by (30°/core/45°). Firstly, the effect of $w_{C}$ is investigated, by keeping constant the value of $w_{F}=0.80$, in the interval $w_{C}\in \left[0,0.40\right]$.

**Figure 9:**
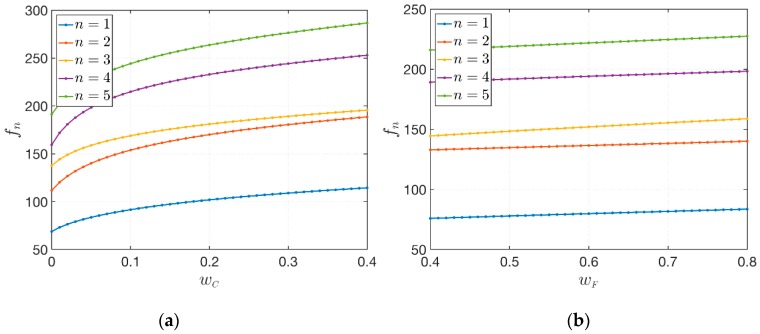
Variation of the first five natural frequencies $f_{n}$ [Hz], for n=1,…,5, of sandwich plates with a damaged soft-core for varying mechanical properties of the constituents: (**a**) Variation of CNT mass fraction; (**b**) Variation of fiber mass fraction.

It should be recalled that the polymer matrix is strengthened by only straight fibers if $w_{C}=0$. Then, the opposite situation is taken into account. The variation of $w_{F}\in \left[0.40,0.80\right]$ is studied for a fixed value of $w_{C}=0.05$. The results are shown in Figure 9 for the first five natural frequencies. The following observations can be deduced:

- The influence of the CNT mass fraction $w_{C}$ is greater than the corresponding variation of the fiber mass fraction $w_{F}$;
- For a small increase of $w_{C}$ next to zero, the variation in terms of natural frequencies that can be obtained is relevant and the behavior is non-linear;
- On the other hand, bigger increases of $w_{F}$ do not produce the same variation of natural frequencies. The behavior is linear in this case.

### 4.7. Discussion on the Mode Shapes

Finally, a brief discussion on the dependency of the previous parameters on the mode shapes is presented. For this purpose, the same geometric features of the previous tests are considered. As far as the mechanical properties of the constituents are concerned, the plate is characterized by $w_{C}=0.05$ and $w_{F}=0.80$. Several configurations are analyzed in order to investigate the effect of the stacking sequence, of the damage and of the exponent of the through-the-thickness distribution of the fibers. The Scheme 3 depicted in Figure 5 is considered here, but similar results in terms of mode shapes could be obtained also with the other schemes. The cases under considerations are summarized in Table 5 for conciseness purposes.

**Figure 10:**
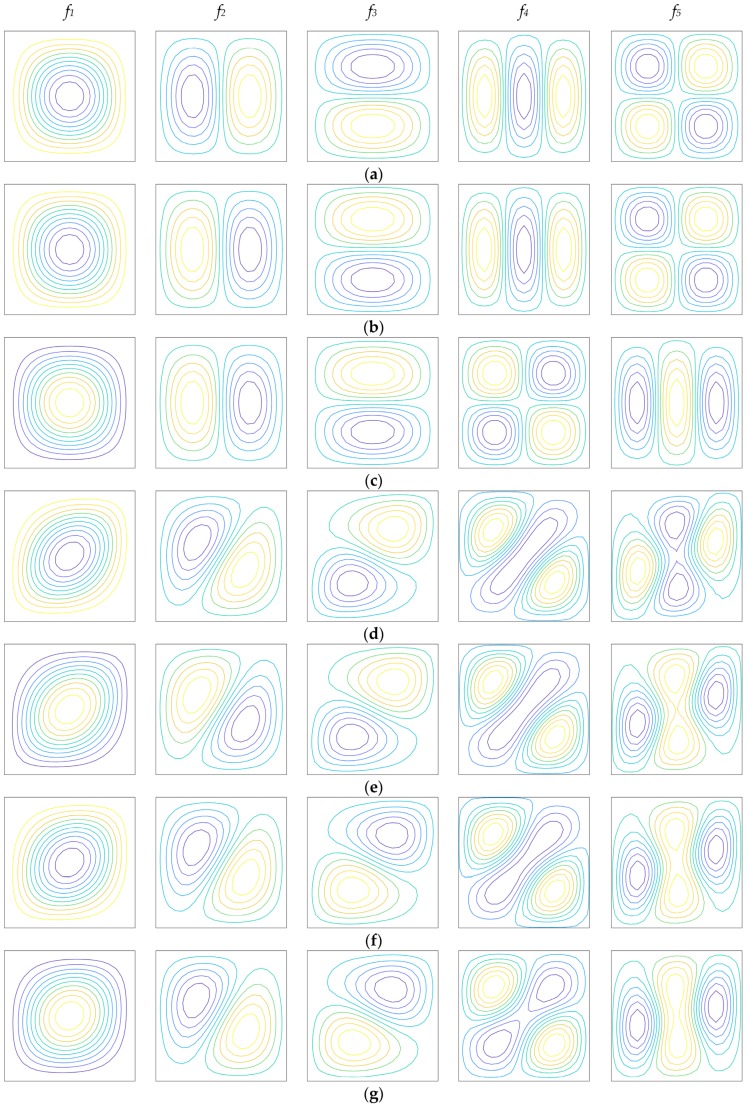
First five mode shapes of the mechanical configurations defined in Table 4: (**a**) Case 1; (**b**) Case 2; (**c**) Case 3; (**d**) Case 4; (**e**) Case 5; (**f**) Case 6; (**g**) Case 7.

The contour plots of the first five mode shapes related to the corresponding natural frequencies are shown in Figure 10 for the seven cases introduced in Table 5. The examples analyzed in this paragraph allow to deduce the following considerations:

- In general, the increase of the damage parameter D does not cause any variation of the mode shapes;
- The mode shapes are highly affected by the orientation of the reinforcing fibers and by the stacking sequence. This aspect can be noted by comparing the same configurations in terms of D and α, but characterized by different lamination schemes (Case 1 and Case 4, for instance);
- As stated in the previous paragraphs, the increase of the exponent α reduces the influence of the reinforcing straight fibers. Therefore, the anisotropic behavior of a laminate with a general stacking sequence can be decreased. For example, the mode shapes of Case 7 tend to the ones related to Case 3 for α=12, even if they are characterized by different fiber orientations;
- The presence of a thicker isotropic core is predominant in the modal amplitudes and only noticeably variations of these mechanical parameters can define some changes in the mode shapes.

## 5. Conclusions

A set of numerical investigations has been presented to describe the mechanical behavior of laminated sandwich plates with a damaged soft-core. The FE formulation has been developed by using a nine-node quadratic rectangular Lagrange element, whereas the theoretical model for laminated plates based on the Reissner-Mindlin model has been enriched by introducing the Murakami’s function. As a consequence, a FE plate theory with 7 degrees of freedom per node has been presented. The external face-sheets are made of composite materials: The polymer matrix has been strengthened by randomly oriented CNTs and oriented straight fibers. Their mechanical characterization has been carried out by using a three-phase model, which include the Eshelby-Mori-Tanaka scheme and the Halpin-Tsai approach. Several parametric tests have been performed to analyze the effect of the damage, the through-the-thickness distribution of the reinforcing fibers, the orientation of the fibers, the stacking sequence, and the mechanical features. The main achievements of the paper are summarized below:

- The use of the Murakami’s function is required to capture the effective mechanical behavior of sandwich structures with an inner soft-core. This aspect is very important especially if a FE commercial code is employed. In fact, it should be recalled that this function is not embedded in plate/shell formulations. Therefore, the results that can be obtained in these circumstances could be inaccurate, unless a 3D-FE modelling is pursued. Nevertheless, this approach is onerous in terms of computational time and resources;
- A non-uniform distribution of the fibers along the thickness of the face-sheets could be employed to model the effective distribution of the reinforcing phase that could occur during the manufacturing process or during the structural life. This research prove that the mechanical response is affected by this parameter;
- A progressive damage in the core causes a corresponding decrease of the natural frequencies, which becomes faster and faster for higher values of damages. The reinforcing layers could recover this situation. If a three-phase composite material is employed to this aim, the design of such layers could be carried out taking into account two parameters, which are the mass fractions of both CNTs and fibers. Nevertheless, a small increase of the CNT mass fraction can cause a quicker and more remarkable variation of the fundamental frequency with respect to the one that could be obtained by controlling the mass fraction of the straight fibers;
- The optimal structural response can be also obtained by choosing accurately the in-plane orientation of the straight fibers. The stacking sequence, in fact, affects the value of the natural frequencies, as well as of the mode shapes.

These comments should be taken into account during the analysis of the mechanical behavior of sandwich structures subjected to a progressive damage, as well as during the process manufacturing if an optimal design has to be pursued.

## Figures and Tables

**Table 1:** Mechanical properties of a single CNT fiber.

Hill’s Elastic Moduli	Density
$k_{C}=271\;\mathrm{GPa}$	$\rho_{C}=1400\;\mathrm{kg}/m^{3}$
$l_{C}=88\;\mathrm{GPa}$	
$m_{C}=17\;\mathrm{GPa}$	
$n_{C}=1089\;\mathrm{GPa}$	
$p_{C}=442\;\mathrm{GPa}$	

**Table 2:** Mechanical properties of the layer constituents.

Constituent	Young’s Moduli	Shear Moduli	Poisson’s Ratios	Density
**Carbon fibers**	$E_{11}^{F}=230\;\mathrm{GPa}$	$G_{12}^{F}=50\;\mathrm{GPa}$	$\nu_{12}^{F}=0.20$	$\rho_{F}=1800\;\mathrm{kg}/m^{3}$
	$E_{22}^{F}=15\;\mathrm{GPa}$		$\nu_{23}^{F}=0.25$	
**Epoxy resin**	$E_{M}=3.27\;\mathrm{GPa}$	−	$\nu_{M}=0.38$	$\rho_{M}=1200\;\mathrm{kg}/m^{3}$

**Table 3:** First ten natural frequencies of a sandwich plate with an inner soft-core for three different lamination schemes: comparison with the 3D-FE solution.

Mode	3D-FE $N_{dofs}=29673$	RMZ $N_{dofs}=3087$	RM $N_{dofs}=2205$	%diff (RMZ)	%diff (RM)
Lamination scheme: (0°/core/0°)					
1	142.126	137.126	169.127	3.52%	19.00%
2	195.625	189.516	224.819	3.12%	14.92%
3	289.303	280.546	324.708	3.03%	12.24%
4	303.824	288.429	408.390	5.07%	34.42%
5	346.073	329.618	453.375	4.75%	31.01%
6	414.415	401.529	463.476	3.11%	11.84%
7	421.211	402.429	533.774	4.46%	26.72%
8	498.657	469.706	635.351	5.81%	27.41%
9	527.513	504.420	650.839	4.38%	23.38%
10	534.573	505.028	678.379	5.53%	26.90%
Lamination scheme: (30°/core/45°)					
1	129.776	125.889	147.134	2.99%	13.38%
2	211.140	203.739	244.133	3.51%	15.63%
3	265.283	253.703	327.302	4.37%	23.38%
4	299.738	288.099	354.309	3.88%	18.21%
5	371.903	354.527	462.224	4.67%	24.29%
6	398.033	381.561	476.960	4.14%	19.83%
7	426.714	404.619	562.840	5.18%	31.90%
8	483.191	459.287	616.601	4.95%	27.61%
9	506.505	484.458	616.724	4.35%	21.76%
10	541.210	512.228	704.809	5.36%	30.23%
Lamination scheme: (−45°/core/45°)					
1	107.180	105.297	112.930	1.76%	5.36%
2	206.687	201.053	225.234	2.73%	8.97%
3	206.687	201.516	225.234	2.50%	8.97%
4	290.914	281.838	322.709	3.12%	10.93%
5	346.351	334.314	392.846	3.48%	13.42%
6	348.741	337.043	395.240	3.35%	13.33%
7	419.280	403.125	479.272	3.85%	14.31%
8	419.280	404.002	479.272	3.64%	14.31%
9	515.903	494.071	608.811	4.23%	18.01%
10	515.903	496.715	608.811	3.72%	18.01%

**Table 4:** Comparison of the frequency parameter ϖ for a two phase fully clamped plate.

Mode	Lei et al. [71] (kp-Ritz)	Lei et al. [71] (Commercial FE)	RMZ (MIX)	RMZ (HT)
Case 1				
1	16.667	16.707	16.671	17.383
2	22.138	22.253	22.098	23.381
3	32.237	32.378	32.211	33.630
4	32.424	32.857	32.242	34.397
5	35.674	35.809	35.652	37.437
6	37.367	37.447	37.403	40.615
Case 2				
1	18.045	18.083	18.055	19.071
2	23.498	23.606	23.448	25.152
3	33.915	34.338	33.712	36.116
4	34.361	34.467	34.324	36.449
5	37.367	37.447	37.403	39.892
6	37.693	37.786	37.637	40.616

**Table 5:** Mechanical configurations investigated to show the variation of the mode shapes due to the stacking sequence, the damage parameter and the exponent of the reinforcing fiber distribution.

Case	Stacking Sequence	Damage D	Exponent α
**1**	(0°/core/0°)	0.00	1
2	(0°/core/0°)	0.50	1
3	(0°/core/0°)	0.50	12
4	(30°/core/45°)	0.00	1
5	(30°/core/45°)	0.50	1
6	(30°/core/45°)	0.50	4
7	(30°/core/45°)	0.50	12

## Appendix A

The submatrices $K_{ij}^{\left(e\right)}$, for i,j=1,…,7, which appear in the element stiffness matrix $K^{\left(e\right)}$ defined in (36), can be computed using the following definitions. The submatrices are presented below row-by-row

(A1) $$\begin{array}{l} K_{11}^{}=\int_{x}\int_{y}\left(B_{x}^{T}\left(A_{11}B_{x}^{}+A_{16}B_{y}^{}\right)+B_{y}^{T}\left(A_{16}B_{x}^{}+A_{66}B_{y}^{}\right)\right)dxdy\\ K_{12}^{}=\int_{x}\int_{y}\left(B_{x}^{T}\left(A_{12}B_{y}^{}+A_{16}B_{x}^{}\right)+B_{y}^{T}\left(A_{26}B_{y}^{}+A_{66}B_{x}^{}\right)\right)dxdy\\ K_{13}=0\\ K_{14}^{}=\int_{x}\int_{y}\left(B_{x}^{T}\left(B_{11}B_{x}^{}+B_{16}B_{y}^{}\right)+B_{y}^{T}\left(B_{16}B_{x}^{}+B_{66}B_{y}^{}\right)\right)dxdy\\ K_{15}^{}=\int_{x}\int_{y}\left(B_{x}^{T}\left(B_{12}B_{y}^{}+B_{16}B_{x}^{}\right)+B_{y}^{T}\left(B_{26}B_{y}^{}+B_{66}B_{x}^{}\right)\right)dxdy\\ K_{16}^{}=\int_{x}\int_{y}\left(B_{x}^{T}\left(F_{11}B_{x}^{}+F_{16}B_{y}^{}\right)+B_{y}^{T}\left(F_{16}B_{x}^{}+F_{66}B_{y}^{}\right)\right)dxdy\\ K_{17}^{}=\int_{x}\int_{y}\left(B_{x}^{T}\left(F_{12}B_{y}^{}+F_{16}B_{x}^{}\right)+B_{y}^{T}\left(F_{26}B_{y}^{}+F_{66}B_{x}^{}\right)\right)dxdy \end{array}$$

(A2) $$\begin{array}{l} K_{21}=K_{12}^{T}\\ K_{22}^{}=\int_{x}\int_{y}\left(B_{y}^{T}\left(A_{22}B_{y}^{}+A_{26}B_{x}^{}\right)+B_{x}^{T}\left(A_{26}B_{y}^{}+A_{66}B_{x}^{}\right)\right)dxdy\\ K_{23}=0\\ K_{24}^{}=\int_{x}\int_{y}\left(B_{y}^{T}\left(B_{12}B_{x}^{}+B_{26}B_{y}^{}\right)+B_{x}^{T}\left(B_{16}B_{x}^{}+B_{66}B_{y}^{}\right)\right)dxdy\\ K_{25}^{}=\int_{x}\int_{y}\left(B_{y}^{T}\left(B_{22}B_{y}^{}+B_{26}B_{x}^{}\right)+B_{x}^{T}\left(B_{26}B_{y}^{}+B_{66}B_{x}^{}\right)\right)dxdy\\ K_{26}^{}=\int_{x}\int_{y}\left(B_{y}^{T}\left(F_{12}B_{x}^{}+F_{26}B_{y}^{}\right)+B_{x}^{T}\left(F_{16}B_{x}^{}+F_{66}B_{y}^{}\right)\right)dxdy\\ K_{27}^{}=\int_{x}\int_{y}\left(B_{y}^{T}\left(F_{22}B_{y}^{}+F_{26}B_{x}^{}\right)+B_{x}^{T}\left(F_{26}B_{y}^{}+F_{66}B_{x}^{}\right)\right)dxdy \end{array}$$

(A3) $$\begin{array}{l} K_{31}=K_{13}^{T},\;K_{32}=K_{23}^{T}\\ K_{33}^{}=\int_{x}\int_{y}\left(B_{x}^{T}\left(\kappa A_{\;44}B_{x}^{}+\kappa A_{\;45}B_{y}^{}\right)+B_{y}^{T}\left(\kappa A_{\;45}B_{x}^{}+\kappa A_{\;55}B_{y}^{}\right)\right)dxdy\\ K_{34}^{}=\int_{x}\int_{y}\left(B_{x}^{T}\left(\kappa A_{\;44}\bar{N}\right)+B_{y}^{T}\left(\kappa A_{\;45}\bar{N}\right)\right)dxdy\\ K_{35}^{}=\int_{x}\int_{y}\left(B_{x}^{T}\left(\kappa A_{\;45}\bar{N}\right)+B_{y}^{T}\left(\kappa A_{\;55}\bar{N}\right)\right)dxdy\\ K_{36}=\int_{x}\int_{y}\left(B_{x}^{T}\left(\kappa H_{44}\bar{N}\right)+B_{y}^{T}\left(\kappa H_{45}\bar{N}\right)\right)dxdy\\ K_{37}=\int_{x}\int_{y}\left(B_{x}^{T}\left(\kappa H_{45}\bar{N}\right)+B_{y}^{T}\left(\kappa H_{55}\bar{N}\right)\right)dxdy \end{array}$$

(A4) $$\begin{array}{l} K_{41}=K_{14}^{T},\;K_{42}=K_{24}^{T},\;K_{43}=K_{34}^{T}\\ K_{44}=\int_{x}\int_{y}\left(B_{x}^{T}\left(D_{11}B_{x}^{e}+D_{16}B_{y}^{}\right)+B_{y}^{T}\left(D_{16}B_{x}^{}+D_{66}B_{y}^{}\right)\right)dxdy+\int_{x}\int_{y}{\bar{N}}^{T}\kappa A_{\;44}\bar{N}dxdy\\ K_{45}=\int_{x}\int_{y}\left(B_{x}^{T}\left(D_{12}B_{y}^{}+D_{16}B_{x}^{}\right)+B_{y}^{T}\left(D_{26}B_{y}^{}+D_{66}B_{x}^{}\right)\right)dxdy+\int_{x}\int_{y}{\bar{N}}^{T}\kappa A_{\;45}\bar{N}dxdy\\ K_{46}=\int_{x}\int_{y}\left(B_{x}^{T}\left(G_{11}B_{x}^{}+G_{16}B_{y}^{}\right)+B_{y}^{T}\left(G_{16}B_{x}^{}+G_{66}B_{y}^{}\right)\right)dxdy+\int_{x}\int_{y}{\bar{N}}^{T}\kappa H_{44}\bar{N}dxdy\\ K_{47}=\int_{x}\int_{y}\left(B_{x}^{T}\left(G_{12}B_{y}^{}+G_{16}B_{x}^{}\right)+B_{y}^{T}\left(G_{26}B_{y}^{}+G_{66}B_{x}^{}\right)\right)dxdy+\int_{x}\int_{y}{\bar{N}}^{T}\kappa H_{45}\bar{N}dxdy \end{array}$$

(A5) $$\begin{array}{l} K_{51}=K_{15}^{T},\;K_{52}=K_{25}^{T},\;K_{53}=K_{35}^{T},\;K_{54}=K_{45}^{T}\\ K_{55}=\int_{x}\int_{y}\left(B_{y}^{T}\left(D_{22}B_{y}^{}+D_{26}B_{x}^{}\right)+B_{x}^{T}\left(D_{26}B_{y}^{}+D_{66}B_{x}^{}\right)\right)dxdy+\int_{x}\int_{y}{\bar{N}}^{T}\kappa A_{\;55}\bar{N}dxdy\\ K_{56}=\int_{x}\int_{y}\left(B_{y}^{T}\left(G_{12}B_{x}^{}+G_{26}B_{y}^{}\right)+B_{x}^{T}\left(G_{16}B_{x}^{}+G_{66}B_{y}^{}\right)\right)dxdy+\int_{x}\int_{y}{\bar{N}}^{T}\kappa H_{45}\bar{N}dxdy\\ K_{57}=\int_{x}\int_{y}\left(B_{y}^{T}\left(G_{22}B_{y}^{}+G_{26}B_{x}^{}\right)+B_{x}^{T}\left(G_{26}B_{y}^{}+G_{66}B_{x}^{}\right)\right)dxdy+\int_{x}\int_{y}{\bar{N}}^{T}\kappa H_{55}\bar{N}dxdy \end{array}$$

(A6) $$\begin{array}{l} K_{61}=K_{16}^{T},\;K_{62}=K_{26}^{T},\;K_{63}=K_{36}^{T},\;K_{64}=K_{46}^{T},\;K_{65}=K_{56}^{T}\\ K_{66}=\int_{x}\int_{y}\left(B_{x}^{T}\left(L_{11}B_{x}^{}+L_{16}B_{y}^{}\right)+B_{y}^{T}\left(L_{16}B_{x}^{}+L_{66}B_{y}^{}\right)\right)dxdy+\int_{x}\int_{y}{\bar{N}}^{T}\kappa P_{44}\bar{N}dxdy\\ K_{67}=\int_{x}\int_{y}\left(B_{x}^{T}\left(L_{12}B_{y}^{}+L_{16}B_{x}^{}\right)+B_{y}^{T}\left(L_{26}B_{y}^{}+L_{66}B_{x}^{}\right)\right)dxdy+\int_{x}\int_{y}{\bar{N}}^{T}\kappa P_{45}\bar{N}dxdy \end{array}$$

(A7) $$\begin{array}{l} K_{71}=K_{17}^{T},\;K_{72}=K_{27}^{T},\;K_{73}=K_{37}^{T},\;K_{74}=K_{47}^{T},\;K_{75}=K_{57}^{T},\;K_{76}=K_{67}^{T}\\ K_{77}=\int_{x}\int_{y}\left(B_{y}^{T}\left(L_{22}B_{y}^{}+L_{26}B_{x}^{}\right)+B_{x}^{T}\left(L_{26}B_{y}^{}+L_{66}B_{x}^{}\right)\right)dxdy+\int_{x}\int_{y}{\bar{N}}^{T}\kappa P_{55}\bar{N}dxdy \end{array}$$

Analogously, the submatrices $M_{ij}^{\left(e\right)}$, for i,j=1,…,7, which appear in the element mass matrix $M^{\left(e\right)}$ introduced in (37), are defined below. The submatrices are presented row-by-row as in the previous case

(A8) $$M_{11}=\int_{x}\int_{y}{\bar{N}}^{T}I_{0}\bar{N}\;dxdy,\;M_{14}=\int_{x}\int_{y}{\bar{N}}^{T}I_{1}\bar{N}\;dxdy,\;M_{16}=\int_{x}\int_{y}{\bar{N}}^{T}I_{3}\bar{N}\;dxdy$$

(A9) $$M_{22}=\int_{x}\int_{y}{\bar{N}}^{T}I_{0}\bar{N}\;dxdy,\;M_{25}=\int_{x}\int_{y}{\bar{N}}^{T}I_{1}\bar{N}\;dxdy,\;M_{27}=\int_{x}\int_{y}{\bar{N}}^{T}I_{3}\bar{N}\;dxdy$$

(A10) $$M_{33}=\int_{x}\int_{y}{\bar{N}}^{T}I_{0}\bar{N}\;dxdy$$

(A11) $$M_{41}=M_{14}^{T},\;M_{44}=\int_{x}\int_{y}{\bar{N}}^{T}I_{2}\bar{N}\;dxdy,\;M_{46}=\int_{x}\int_{y}{\bar{N}}^{T}I_{4}\bar{N}\;dxdy$$

(A12) $$M_{52}=M_{25}^{T},\;M_{55}=\int_{x}\int_{y}{\bar{N}}^{T}I_{2}\bar{N}\;dxdy,\;M_{57}=\int_{x}\int_{y}{\bar{N}}^{T}I_{4}\bar{N}\;dxdy$$

(A13) $$M_{61}=M_{16}^{T},\;M_{64}=M_{46}^{T},\;M_{66}=\int_{x}\int_{y}{\bar{N}}^{T}I_{5}\bar{N}\;dxdy$$

(A14) $$M_{72}=M_{27}^{T},\;M_{75}=M_{57}^{T},\;M_{77}=\int_{x}\int_{y}{\bar{N}}^{T}I_{5}\bar{N}\;dxdy$$

The null submatrices ($M_{ij}^{\left(e\right)}=0$) are omitted in definitions (A8)–(A14). The full integration scheme is always used to compute the elements of the mass matrix.

It can be easily noted from definitions (A1)–(A14) that the stiffness and mass matrices are both symmetrical operators, thus $K_{ij}^{\left(e\right)}=K_{ji}^{\left(e\right)},\;M_{ij}^{\left(e\right)}=M_{ji}^{\left(e\right)}$. It should be recalled that the same definitions $K_{ij}^{\left(e\right)},\;M_{ij}^{\left(e\right)}$ are valid also for the well-known RM theory, for i,j=1,…,5.

## References

[1] Duncan W.J., Collar A.R. (1934). A method for the solution of oscillations problems by matrices. Phil. Mag..

[2] Duncan W.J., Collar A.R. (1935). Matrices applied to the motions of damped systems. Phil. Mag..

[3] Hrennikoff A. (1941). Solution of Problems of Elasticity by the Frame–Work Method. ASME J. Appl. Mech..

[4] Courant R. (1943). Variational methods for the solution of problems of equilibrium and vibration. B. Am. Math. Soc..

[5] Clough R.W. The finite element method in plane stress analysis. Proceedings of the 2nd ASCE conference in electronics computation.

[6] Melosh R.J. (1963). Basis for derivation of matrices for the direct stiffness method. AIAA J..

[7] Oden J.T. (1972). Finite Elements of Nonlinear Continua.

[8] Oden J.T., Reddy J.N. (1976). An Introduction to the Mathematical Theory of Finite Elements.

[9] Hinton E. (1988). Numerical Methods and Software for Dynamic Analysis of Plates and Shells.

[10] Zienkiewicz O.C. (1991). The Finite Element Method.

[11] Reddy J.N. (1993). An Introduction to the Finite Element Method.

[12] Hughes T.J.R. (2000). The Finite Element Method-Linear Static and Dynamic Finite Element Analysis.

[13] Ferreira A.J.M. (2008). MATLAB Codes for Finite Element Analysis.

[14] Martínez-Pañeda E. (2019). On the finite element implementation of functionally graded materials. Materials.

[15] Nguyen H.N., Nguyen T.Y., Tran K.V., Tran T.T., Nguyen T.T., Phan V.D., Do T.V. (2019). A finite element model for dynamic analysis of triple-layer composite plates with layers connected by shear connectors subjected to moving load. Materials.

[16] Leonetti L., Fantuzzi N., Trovalusci P., Tornabene F. (2019). Scale effects in orthotropic composite assemblies as micropolar continua: A comparison between weak-and strong-form finite element solutions. Materials.

[17] Liu P., Bui T.Q., Zhu D., Yu T.T., Wang J.W., Yin S.H., Hirose S. (2015). Buckling failure analysis of cracked functionally graded plates by a stabilized discrete shear gap extended 3-node triangular plate element. Compos. Part B Eng..

[18] Hosseini S.S., Bayesteh H., Mohammadi S. (2013). Thermo-mechanical XFEM crack propagation analysis of functionally graded materials. Mat. Sci. Eng. A.

[19] Yin S., Yu T., Bui T.Q., Liu P., Hirose S. (2016). Buckling and vibration extended isogeometric analysis of imperfect graded Reissner-Mindlin plates with internal defects using NURBS and level sets. Comput. Struct..

[20] Singh S.K., Singh I.V., Mishra B.K., Bhardwaj G., Singh S.K. (2018). Analysis of cracked plate using higher-order shear deformation theory: Asymptotic crack-tip fields and XIGA implementation. Comput. Method. Appl. M..

[21] Lemaitre J., Chaboche J.L. (1990). Mechanics of Solid Materials.

[22] Reddy J.N., Miravete A. (1995). Practical Analysis of Composite Laminates.

[23] Tarantino A.M. (2014). Equilibrium paths of a hyperelastic body under progressive damage. J. Elast..

[24] Lanzoni L., Tarantino A.M. (2014). Damaged hyperelastic membranes. Int. J. Nonlinear Mech..

[25] Lanzoni L., Tarantino A.M. (2015). Equilibrium configurations and stability of a damaged body under uniaxial tractions. Z. Angew. Math. Phys..

[26] Lanzoni L., Tarantino A.M. (2016). A simple nonlinear model to simulate the localized necking and neck propagation. Int. J. Nonlinear Mech..

[27] Savino V., Lanzoni L., Tarantino A.M., Viviani M. (2018). Simple and effective models to predict the compressive and tensile strength of HPFRC as the steel fiber content and type changes. Compos. Part B Eng..

[28] Falope F.O., Lanzoni L., Tarantino A.M. (2018). Modified hinged beam test on steel fabric reinforced cementitious matrix (SFRCM). Compos. Part B Eng..

[29] Dezi L., Menditto G., Tarantino A.M. (1990). Homogeneous structures subjected to successive structural system changes. J. Eng. Mech. ASCE.

[30] Dezi L., Tarantino A.M. (1991). Time dependent analysis of concrete structures with variable structural system. ACI Mater. J..

[31] Dezi L., Menditto G., Tarantino A.M. (1992). Viscoelastic heterogeneous structures with variable structural system. J. Eng. Mech. ASCE.

[32] Dezi L., Tarantino A.M. (1993). Creep in continuous composite beams. Part II: Parametric study. J. Eng. Mech. ASCE.

[33] Tarantino A.M. (2008). Homogeneous equilibrium configurations of a hyperelastic compressible cube under equitriaxial dead-load tractions. J. Elast..

[34] Vinson J.R. (1993). The Behavior of Shells Composed of Isotropic and Composite Materials.

[35] Jones R.M. (1999). Mechanics of Composite Materials.

[36] Reddy J.N. (2004). Mechanics of Laminated Composite Plates and Shells-Theory and Analysis.

[37] Barbero E.J. (2011). Introduction to Composite Materials Design.

[38] Tornabene F., Bacciocchi M., Fantuzzi N., Reddy J.N. (2019). Multiscale approach for three-phase cnt/polymer/ fiber laminated nanocomposite structures. Polym. Compos..

[39] Bacciocchi M., Tarantino A.M. (2019). Time-dependent behavior of viscoelastic three-phase composite plates reinforced by carbon nanotubes. Compos. Struct..

[40] Popov V.N., Van Doren V.E. (2000). Elastic properties of single-walled carbon nanotubes. Phys. Rev. B.

[41] Qian D., Wagner G.J., Liu W.K., Yu M.F., Ruoff R.S. (2002). Mechanics of carbon nanotubes. Appl. Mech. Rev..

[42] Fidelus J.D., Wiesel E., Gojny F.H., Schulte K., Wagner H.D. (2005). Thermo–mechanical properties of randomly oriented carbon/epoxy nanocomposites. Compos. Part A Appl. S..

[43] Ray M.C., Batra R.C. (2009). Effective Properties of Carbon Nanotube and Piezoelectric Fiber Reinforced Hybrid Smart Composites. J. App. Mech. T. ASME.

[44] Song Y.S., Youn J.R. (2006). Modeling of effective elastic properties for polymer based carbon nanotube composites. Polymer.

[45] Coiai S., Passaglia E., Pucci A., Ruggeri G. (2015). Nanocomposites based on thermoplastic polymers and functional nanofiller for sensor applications. Materials.

[46] Bhattacharya M. (2016). Polymer Nanocomposites-a comparison between carbon nanotubes, graphene, and clay as nanofillers. Materials.

[47] Acierno S., Barretta R., Luciano R., Marotti de Sciarra F., Russo P. (2017). Experimental evaluations and modeling of the tensile behavior of polypropylene/single-walled carbon nanotubes fibers. Compos. Struct..

[48] Wang G., Wang Y., Luo Y., Luo S. (2018). Carbon nanomaterials based smart fabrics with selectable characteristics for in–line monitoring of high-performance composites. Materials.

[49] Arena M., Viscardi M., Barra G., Vertuccio L., Guadagno L. (2019). Multifunctional performance of a nano–modified fiber reinforced composite aeronautical panel. Materials.

[50] Odegard G.M., Gates T.S., Wise K.E., Park C., Siochi E.J. (2003). Constitutive modeling of nanotube–reinforced polymer composites. Compos. Sci. Technol..

[51] Shi D.L., Huang Y.Y., Hwang K.C., Gao H. (2004). The effect of nanotube waviness and agglomeration on the elastic property of carbon nanotube–reinforced composites. J. Eng. Mater. T. ASME.

[52] Eshelby J.D. (1957). The determination of the elastic field of an ellipsoidal inclusion, and related problems. P. Roy. Soc. Lond. A Mat..

[53] Mori T., Tanaka K. (1973). Average stress in matrix and average elastic energy of materials with misfitting inclusions. Acta Metall..

[54] Safaei B., Moradi-Dastjerdi R., Qin Z., Behdinan K., Chu F. (2019). Determination of thermoelastic stress wave propagation in nanocomposite sandwich plates reinforced by clusters of carbon nanotubes. J. Sandw. Struct. Mater..

[55] Halpin J.C. (1969). Effects of Environmental Factors on Composite Materials.

[56] Tsai S.W. (1964). Structural Behavior of Composite Materials.

[57] Tsai S.W. (1965). Strength Characteristics of Composite Materials.

[58] Hill R. (1964). Theory of mechanical properties of fibre-strengthened materials: I. Elastic behavior. J. Mech. Phys. Solids.

[59] Hill R. (1964). Theory of mechanical properties of fibre–strengthened materials: II. Inelastic behavior. J. Mech. Phys. Solids.

[60] Thostenson E.T., Li W.Z., Wang D.Z., Ren Z.F., Chou T.W. (2002). Carbon nanotube/carbon fiber hybrid multiscale composites. J. Appl. Phys..

[61] Bekyarova E., Thostenson E.T., Yu A., Kim H., Gao J., Tang J., Hahn H.T., Chou T.W., Itkis M.E., Haddon R.C. (2007). Multiscale carbon nanotube-carbon fiber reinforcement for advanced epoxy composites. Langmuir.

[62] Kim M., Park Y.B., Okoli O.I., Zhang C. (2009). Processing, characterization, and modeling of carbon nanotube-reinforced multiscale composites. Compos. Sci. Technol..

[63] Rafiee M., He X.Q., Mareishi S., Liew K.M. (2014). Modeling and stress analysis of smart CNTs/fiber/polymer multiscale composite plates. Int. J. Appl. Mech..

[64] Chamis C.C., Sendeckyj G.P. (1968). Critique on theories predicting thermoelastic properties of fibrous composites. J. Compos. Mater..

[65] Hill R. (1964). Theory of mechanical properties of fibre–strengthened materials: III. Self–consistent model. J. Mech. Phys. Solids.

[66] Chou T.W. (1980). A self-consistent approach to the elastic stiffness of short–fiber composites. J. Compos. Mater..

[67] Hashin Z. (1962). The elastic moduli of heterogeneous materials. J. Appl. Mech. T. ASME.

[68] Hashin Z., Rosen B.W. (1964). The elastic moduli of fiber-reinforced materials. J. Appl. Mech. T. ASME.

[69] Ekvall J.C. (1961). Elastic Properties of Orthotropic Monofilament Laminates.

[70] Chen C.H., Cheng S. (1967). Mechanical properties of fiber reinforced composites. J. Compos. Mater..

[71] Lei Z.X., Liew K.M., Yu J.L. (2013). Free vibration analysis of functionally graded carbon nanotube-reinforced composite plates using the element-free kp-Ritz method in thermal environment. Compos Struct..

[72] Gusella F., Cluni F., Gusella V. (2019). Homogenization of dynamic behaviour of heterogeneous beams with random Young’s modulus. Eur. J. Mech. A Solid..

[73] Gusella F., Cluni F., Gusella V. (2019). Homogenization of the heterogeneous beam dynamics: The influence of the random Young’s modulus mixing law. Compos. Part B Eng.

[74] Reddy J.N., Chin C.D. (1998). Thermomechanical analysis of functionally graded cylinders and plates. J. Therm. Stresses.

[75] Reddy J.N. (2000). Analysis of functionally graded plates. Int. J. Numer. Meth. Engng..

[76] Vel S.S., Batra R.C. (2004). Three-dimensional exact solution for the vibration of functionally graded rectangular plates. J. Sound Vib..

[77] Batra R.C., Jin J. (2005). Natural frequencies of a functionally graded rectangular plate. J. Sound Vib..

[78] Kim J., Reddy J.N. (2015). A general third-order theory of functionally graded plates with modified couple stress effect and the von Kármán nonlinearity: Theory and finite element analysis. Acta Mech..

[79] Kim J., Reddy J.N. (2017). Modeling of functionally graded smart plates with gradient elasticity effects. Mech. Adv. Mater. Struct..

[80] Alexandrov S., Wang Y.C., Lang L. (2019). A theory of elastic/plastic plane strain pure bending of FGM sheets at large strain. Materials.

[81] Tornabene F. (2009). Free vibration analysis of functionally graded conical, cylindrical shell and annular plate structures with a four-parameter power-law distribution. Comput. Method. Appl. Mech. Eng..

[82] Tornabene F., Viola E. (2009). Free vibration analysis of functionally graded panels and shells of revolution. Meccanica.

[83] Sofiyev A.H., Kuruoglu N. (2015). Dynamic instability of three-layered cylindrical shells containing an FGM interlayer. Thin Wall. Struct..

[84] Alibeigloo A. (2016). Thermo elasticity solution of sandwich circular plate with functionally graded core using generalized differential quadrature method. Compos. Struct..

[85] Civalek Ö., Baltacıoglu A.K. (2019). Free vibration analysis of laminated and FGM composite annular sector plates. Compos. Part B Eng..

[86] Nguyen H.N., Tan T.C., Luat D.T., Phan V.D., Thom D.V., Minh P.V. (2019). Research on the buckling behavior of functionally graded plates with stiffeners based on the third–order shear deformation theory. Materials.

[87] Lanc D., Vo T.P., Turkalj G., Lee J. (2015). Buckling analysis of thin–walled functionally graded sandwich box beams. Thin Wall. Struct..

[88] Lanc D., Turkalj G., Vo T., Brnic J. (2016). Nonlinear buckling behaviours of thin-walled functionally graded open section beams. Compos. Struct..

[89] Kim J., Zur K.K., Reddy J.N. (2019). Bending, free vibration, and buckling of modified couples stress-based functionally graded porous micro-plates. Compos. Struct..

[90] Barretta R., Feo L., Luciano R., Marotti de Sciarra F., Penna R. (2016). Functionally graded Timoshenko nanobeams: A novel nonlocal gradient formulation. Compos. Part B Eng..

[91] Apuzzo A., Barretta R., Faghidian S.A., Luciano R., Marotti de Sciarra F. (2019). Nonlocal strain gradient exact solutions for functionally graded inflected nano-beams. Compos. Part B Eng..

[92] Carrera E. (1996). C^o^ reissner-mindlin multilayered plate elements including Zig-Zag and interlaminar stress continuity. Int. J. Numer. Meth. Eng..

[93] Carrera E. (2001). Developments, ideas and evaluations based upon the Reissner’s mixed theorem in the modeling of multilayered plates and shells. Appl. Mech. Rev..

[94] Carrera E. (2003). Historical review of Zig-Zag theories for multilayered plates and shells. Appl. Mech. Rev..

[95] Carrera E. (2003). Theories and finite elements for layered plates and shells: A unified compact formulation with numerical assessment and benchmarking. Arch. Comput. Meth. Eng..

[96] Carrera E. (2004). On the use of the Murakami’s Zig-Zag function in the modeling of layered plates and shells. Comput. Struct..

[97] Maturi D.A., Ferreira A.J.M., Zenkour A.M., Mashat D.S. (2013). Analysis of laminated shells by murakami’s Zig–Zag theory and radial basis functions collocation. J. Appl. Math..

[98] Brischetto S., Carrera E., Demasi L. (2009). Improved bending analysis of sandwich plates using a Zig–Zag function. Compos. Struct..

[99] Hu H., Belouettar S., Daya E.M., Potier-Ferry M. (2006). Evaluation of kinematic formulations for viscoelastically damped sandwich beam modeling. J. Sandw. Struct. Mater..

[100] Murakami H. (1986). Laminated composite plate theory with improved in-plane responses. J. Appl. Mech..

[101] Lenci S., Tarantino A.M. (1996). Chaotic dynamics of an elastic beam resting on a Winkler–type soil. Chaos Soliton. Fract..

